# Paper to Pixels: Enhancing Unilateral Neglect Assessment Using the New Computer Vision-Based Tool CANDO

**DOI:** 10.3390/brainsci16050541

**Published:** 2026-05-21

**Authors:** Lisa Beckmann, Rylan Donohoe, Doris Schmid, Ines C. Kiphuth, Karin Ludwig, Thomas Schenk

**Affiliations:** 1 Clinical Neuropsychology, Department of Psychology, Ludwig-Maximilians-Universität München, 80802 Munich, Germany; l.beckmann@psy.lmu.de (L.B.); rylan.donohoe@mail.mcgill.ca (R.D.); doris.schmid@psy.lmu.de (D.S.); karin.ludwig@psy.lmu.de (K.L.); 2 Department of Mathematics and Statistics, McGill University, Montreal, QC H3A 0G4, Canada; 3 Zentrum für Neurologie, Klinikum am Europakanal, 91056 Erlangen, Germany; christine.kiphuth@bezirkskliniken-mfr.de

**Keywords:** unilateral neglect, spatial neglect, neuropsychological assessment, behavioural inattention test, subjective scoring, computer vision

## Abstract

**Background/Objectives**: The main aim of this article is to introduce a novel tool that allows the automatic scoring of many of the subtests from the conventional subpart of the Behavioural Inattention Test (BIT) and its German adaptation, the Neglect Test (NET). BIT and NET are standard test batteries used in the diagnosis of neglect. Our article has two parts. First, we examine the shortcomings of manual scoring, and secondly, we introduce our computer vision tool and evaluate its diagnostic validity and efficacy. **Methods**: In Part 1, diagnostic consistency was examined across raters with varying expertise using selected BIT and NET tasks, with repeated assessments conducted under controlled evaluation conditions. In Part 2, a computer vision-based tool (CANDO) was developed to automate scoring using a deterministic computer vision pipeline designed to reproducibly apply scoring criteria across tasks. The performance of CANDO was compared with ground truth across cancellation, line bisection, and copying tasks. **Results**: Manual scoring showed high overall agreement between and within raters under ideal conditions. However, diagnostic classification still differed across raters and repeated assessments. These inconsistencies were primarily driven by drawing and copying tasks. CANDO achieved very high accuracy for cancellation and line bisection tasks and strong agreement for copying tasks, while reducing variability associated with subjective judgment, time pressure, and oversight. The remaining discrepancies between computer vision and human raters had limited impact on diagnostic outcomes. **Conclusions**: Manual assessment of unilateral neglect is vulnerable to inconsistencies arising from subjective evaluation and the structural limitations of scoring systems. Computer vision-based automation can reduce diagnostic variability, improve reproducibility, and increase assessment efficiency, while preserving clinically relevant information. The presented framework provides a practical tool to support higher-quality neglect assessment.

## 1. Introduction

Unilateral neglect, hereafter referred to as neglect, typically follows damage to the right hemisphere of the brain [1]. In the acute stage following a stroke, about 34% of all stroke patients show signs of neglect (43% after right-hemisphere damage, 23% after left-hemisphere damage) [2]. Neglect is frequently understood as the lack of awareness of the contralesional side of space, and patients often describe the experience as if one half of their world ceases to exist [1]. Neglect affects a variety of daily activities, such as navigation, reading, and self-care, all of which drastically reduce self-sufficiency and quality of life. Without an appropriate diagnostic procedure and treatment, neglect can lead to prolonged disability and poor rehabilitation outcomes [3,4,5].

Early identification of neglect facilitates planning of rehabilitative interventions and individually tailored therapeutic approaches, which are necessary for achieving the best rehabilitation outcomes [6]. This is especially relevant for the acute phase after a stroke, as early interventions tend to be more effective at reducing long-term consequences. An early diagnosis also helps reduce secondary problems, such as increased dependency on caregivers and reduced functional recovery [7,8]. Symptoms of neglect are often subtle and can vary widely between patients, making its detection difficult for clinicians and researchers. Besides the inattention toward one side of space, more complex forms of neglect can also occur, e.g., an allocentric form in which one side of each object or the patient’s own body is neglected [1].

In light of the subtle and variable presentation of neglect, clinicians and researchers often rely on standardized paper-and-pencil tests to support diagnosis, though these too have notable limitations. Paper-and-pencil tests and observational methods are often subjective and prone to clinician bias. For instance, a rater’s interpretation of a patient’s performance may vary based on their experience [9] and personal expectations [10] about what constitutes a “normal” result. While there is no universally accepted set of diagnostic criteria for neglect, most studies use the Behavioural Inattention Test (BIT) [11] or a variant, such as the Spanish adaptation [12], the German adaptation [13], or the Japanese adaptation [14]. However, different clinical settings may use different test batteries or different subsets of test batteries to assess neglect, resulting in diagnostic variability. The analysis and interpretation of some neglect tests are subjective and rely on clinician judgment (e.g., tasks demanding drawing from memory or copying of templates). These issues further increase the possibility of misinterpretation or inconsistent assessment.

In addition to the difficulties in assigning consistent scores for tests that require subjective judgments, assessing and scoring neglect tests is time consuming. Furthermore, recent suggestions for improving the sensitivity of neglect tests require additional time. Rorden and Karnath [15] developed a so-called center-of-cancellation (CoC) measure—a metric of lateralized bias for cancellation tasks that provides more sensitive and specific detection of neglect signs. Rorden and Karnath provided a digital tool to calculate the CoC, but using this tool still requires an additional time-consuming step to provide the necessary input for computing the CoC [15].

Despite the widespread use of paper-and-pencil tests like the BIT and its German counterpart (NET), important limitations remain insufficiently understood. This is especially true for the subtests requiring qualitative judgment, such as the drawing or copying tasks. Furthermore, no standardized and objective approach currently exists to reduce such variability while maintaining the clinical relevance and practicality of conventional neglect assessment procedures.

One path that has been taken to combat issues with paper-and-pencil procedures is the use of digital approaches to neglect assessment, which can capture additional behavioral measures such as reaction times, visual exploration patterns, gaze behavior, navigation, and motor responses (e.g., [16,17,18]). Existing approaches include computerized and tablet-based adaptations of classical cancellation and line bisection tasks, which allow automated quantitative scoring (e.g., [19,20]) and may improve sensitivity for subtle neglect symptoms, as well as virtual and augmented reality paradigms designed to increase ecological validity by assessing neglect patients during navigation and interaction in simulated environments (e.g., [21,22,23,24]). While these approaches offer important advantages, they also face several practical and methodological limitations, including small validation samples, heterogeneous outcome measures (e.g., [18]), uncertain added diagnostic value relative to established neuropsychological tests, and potential usability problems (e.g., [25]) related to fatigue, cybersickness, visual or motor impairments, cognitive load, unfamiliarity with technology, and cost. Additionally, these approaches cannot rely on the well-established norms of conventionally used tests and many require specialized hardware or fully digital task administration, limiting compatibility with paper-and-pencil-based existing clinical workflows.

In this study, we propose and introduce a computer vision (CV)-based automation approach for analyzing the results of paper-and-pencil tests used in the assessment of neglect symptoms. We hope that such an approach will dramatically reduce the time needed to analyze neglect tests while increasing the objectivity, reproducibility, and consistency of assessments. Furthermore, by providing easy access to additional digitally available analysis procedures, such as the CoC, it may enhance the specificity and sensitivity of conventional neglect tests without requiring further time investment.

Before introducing our CV-based approach, we will first explore and quantify some of the difficulties inherent in the current procedures used to analyze neglect tests. Thus, the first part of our study consists of an empirical examination of the diagnostic shortcomings in the current procedure for evaluating the conventional subpart of the BIT (BIT-c) and its German counterpart, with a particular focus on rating consistency and sources of variability. These findings characterize the problem our CV-based approach aims to address and thus provide a baseline against which the performance of our CV-based procedures can be judged. In the second part of the study, we then introduce and validate our computer vision-based framework, the Computer-based Analysis of Neglect Deficits On paper (CANDO).

## 2. Part 1: Diagnostic Shortcomings in the Evaluation of the BIT-c and Its German Adaptation

The Behavioural Inattention Test was developed to address the limitations of earlier neglect assessments by combining multiple tasks into a single, structured test battery. It consists of two parts: the conventional part (BIT-c) and the behavioral part (BIT-b; [11]). As a starting point for preparing for the CV implementation, we focused on the conventional subpart, i.e., the paper-and-pencil tests. The BIT-c is the most commonly used neglect assessment in research [26]. The BIT-c integrates established measures that had been prominent before its development. For example, the line-crossing subtest is an adaptation of Albert’s task in which patients are asked to cross out all small lines on a test sheet [27].

Even though combining tests into a structured test battery was a major improvement in neglect diagnostics, the BIT-c has several limitations. A recent review by Williams and colleagues [26] showed that, despite the BIT-c providing some of the best available evidence, the quality of evidence for psychometric properties was still low or very low. This is the case for most neglect assessment tools and suggests that the real test properties can deviate substantially from the reported properties [26]. Many patients, especially those with mild deficits or after improvement, score within the normal range, despite showing functional deficits in everyday life [28]. A central obstacle for the diagnostic process is the evaluation method: the BIT-c relies heavily on quantitative measures (e.g., the number of crossed-out targets) and only sparsely integrates qualitative aspects—such as distortions in drawings or the spatial consistency of errors—which require subjective judgment and are a key source of scoring variability [15].

The Neglect Test (NET) is the German adaptation of the BIT, developed to align with linguistic, cultural, and normative differences in the German-speaking population [13]. Most subtests remain the same or have slight changes (e.g., usage of German distractor words); see Table 1. In the figure-and-shape-copying subtest, the second template figure—a Necker cube [29]—was replaced with an equiangular diamond. The authors did not specify their reasoning for this change. Anecdotally, we find that performance in copying the Necker cube is highly related to a patient’s level of education and, due to the figure’s complexity, reflects deficits on multiple cognitive levels. This is further supported by findings of Seki and colleagues linking performance on this task to verbal intelligence [30], as well as by the ongoing discussion surrounding its use in Alzheimer’s disease research [31,32]. Furthermore, the second part of the figure-and-shape-copying subtest, the drawing of geometric shapes, was dropped. Similarly, in the representational drawing task, the NET only includes the drawing of a clock face and omits drawings of a man/woman and a butterfly.

There are several differences between the BIT-c and NET in how subtest scores are calculated and also in how subtest scores are used to compute a total score. In the BIT-c [11], different subtests yield different maximum scores. For example, in cancellation subtests, the score corresponds to the number of crossed-out target symbols, while for the figure-and-shape-copying subtest, fewer points are given and the points reflect the quality of the copy. To compute the total score of the BIT-c, the subtest scores are simply added. This leads to uneven weighting, as subtests with larger maximum scores contribute more heavily to the BIT-c total than those with smaller ones. In contrast, the NET converts raw values into standardized scores (ranging from 0 to 10) for each subtest [13]. This conversion ensures that all subtests in the NET are weighted equally rather than in proportion to their maximum raw scores, as in the BIT-c. This standardization happens separately for each drawing in the figure-and-shape-copying subtest in the NET. Another central difference in the evaluation of the figure-and-shape-copying subtest is that the BIT-c uses a binary criterion per drawing, assigning one point for a complete drawing and zero for an incomplete one [11]. In contrast, the NET uses a scale from zero to three and examines each drawing based on gestalt, presence of details, and spatial arrangement [13].

Furthermore, the BIT-c provides cut-off values for each subtest and the total sum score, indicating the presence or absence of neglect [11]. The NET only provides a cut-off for the total sum score of the standard values, but uses the test scores to distinguish between different levels of neglect severity (no, mild, strong, and severe neglect) [13]. A comprehensive overview of the differences between NET and BIT-c is presented in Table 1.

During the development and publication of the test batteries, both the BIT-c and NET were evaluated for their psychometric properties with studies reporting good reliability. According to the BIT manual [11] and two studies [33,34], inter-rater reliability was tested by comparing the rating results of two raters for 13 partients, demonstrating a strong correlation of r = 0.99. Additionally, test–retest reliability was examined by looking at 10 participants on two separate days, finding a correlation of r = 0.99 between the first and second tests. A more recent study by Hannaford and Grower [35] confirmed relatively high inter-rater reliability (overall intra-class correlation coefficient (ICC) = 0.994). This inter-rater reliability varied strongly between subtests, ranging from ICC = 0.774 for the representational drawing subtest to ICC = 0.999 for the line-crossing subtest [35]. In comparison, the NET manual reports a retest reliability of r = 0.93 for the entire test score with 19 patients at different test dates [13]. The manual also reports subtest-level retest reliabilities, with correlations ranging from r = 0.27 (copying: flower) to r = 0.99 (star cancellation). For inter-rater reliability, they report 95% agreement between two raters on 20 patients for the total score. This agreement varies between 85% (copying: flower, star) and 100% (line crossing, letter cancellation, star cancellation, and line bisection) [13].

While for both batteries the inter-rater reliability and the test-retest reliability were evaluated, no study has looked at the intra-rater reliability, i.e., at how consistent a rater is in evaluating the same patient files twice. While test–retest reliability may already show a tendency for this consistency, it is confounded by patient practice effects and potential remission of neglect between the testing sessions (e.g., 15 days in [11]).

One important distinction in neglect assessment is the use of objective and subjective criteria. All cancellation subtests are scored based on the numerical count of crossed-out targets [11,13] and provide clear, quantifiable data that allow for statistical comparisons. These scores are typically consistent within and between raters and are therefore considered objective. In contrast, representational drawing and figure-and-shape-copying subtests require clinicians to evaluate the accuracy and completeness of patients’ drawings [11,13], which involves qualitative judgment and is thus more susceptible to both inter- and intra-rater variability.

In the present study, we aimed to examine potential problems in the scoring of the BIT-c, particularly issues concerning reliability and consistency in subtests that rely on subjective judgments. More generally, we sought to identify which subtests are more vulnerable to differences in interpretation within and between raters. Based on the differing evaluation criteria across subtests, we expected greater variability for tasks relying on more subjective qualitative judgments, such as evaluating the completeness, closure, or arrangement of drawings, than for tasks based on more objective quantitative criteria, such as crossed-out targets or measuring line bisection deviations.

To address the limitations of previous examinations—which often involved only two raters and did not separately assess consistency between and within raters—we included raters with three levels of expertise (expert clinicians, naïve students with practice, and naïve students without practice), each of whom rated responses from ten patients twice. The patient files spanned the full range of potential responses, from patients with mild neglect who followed instructions perfectly to those who were severely impaired and struggled to comply with instructions. Importantly, these patient files were deliberately selected to include diagnostically straightforward as well as diagnostically challenging response patterns in order to systematically probe potential sources of disagreement in the existing scoring criteria, rather than to provide a representative estimate of rating consistency in routine clinical populations. Furthermore, raters evaluated the material in a blocked, subtest-wise manner rather than as complete patient profiles, to allow subtest-specific measurement of rating times and to minimize the extent to which overall consistency of performance across subtests could influence individual scoring decisions.

With this design, we aimed to overcome several shortcomings of earlier studies. We assessed both inter-rater and intra-rater consistency across a broad range of patient performances and examined the influence of rater expertise on scoring and diagnostic decisions. In addition, we evaluated how diagnostic parameters affect outcomes, incorporated repeated assessments of identical tests by the same rater, and measured both the time required for assessment and raters’ confidence in their judgments. This comprehensive approach allows us to better understand sources of disagreement, their implications for diagnostic validity, and potential paths for improving both research and clinical practice in neglect assessment.

### 2.1. Materials and Methods

#### 2.1.1. Participants

A total of 21 raters took part in the experiment. Fourteen of them were naïve to the process of neuropsychological testing and seven were experts who already had substantial experience with neuropsychological diagnostics and neglect. Three of the authors (LB, DS, and KL) participated in the study as expert raters. Two raters were excluded from the analyses because they disclosed having limited professional experience or experience in a related field after the experiment (psychology student working in a neuropsychology unit in a clinic; psychotherapist in training). Although this experience was relevant, it did not meet our criteria for expert status, and thus they could not be clearly classified as either naïve or expert.

Of the naïve group, nine raters were female and four were male, with a mean age of 24.14 years (SD = 4.89 years). In the expert group, six raters were female and one was male (M = 28.14 years; SD = 6.07 years). The naïve raters and three of the experts received 10 euros per hour or course credit as compensation. The study was approved by the ethics board of the department of psychology at the Ludwig-Maximilians-Universität München. Although seven of the naïve raters were psychology students (in their first semester), none had practical experience with neuropsychological diagnostics or had yet attended lectures on neuropsychology or diagnostics.

All experts had substantial knowledge and practical experience with neuropsychological testing and diagnostic evaluation and were recruited through our clinical neuropsychology lab, our outpatient clinic, and the neurology department of the Klinikum Großhadern located in Munich.

#### 2.1.2. Stimuli and Materials

From the BIT-c (Thames Valley Test Company, Bury St. Edmunds, UK) [11] and NET (Hogrefe Verlag GmbH & Co. KG, Göttingen, Germany) [13] manuals, we selected four subtests: (1) letter cancellation, (2) line bisection, (3) figure and shape copying, and (4) representational drawing. Throughout this manuscript, references to BIT-c and NET refer to these respective manuals unless otherwise stated. These subtests are representative of the three main test types of the BIT-c: cancellation, bisection, and drawing subtests. We included the representational drawing of a clock, as it is a widely used task in neuropsychology in general.

The ten “patients” were constructed as composite cases based on subtests drawn from a sample of over 80 patients, with the aim of representing the wide variability of performance observed in individual patients with brain damage on the BIT-c/NET. Thus, “Patient 1” could have the letter-cancellation responses of one patient, while the figure-and-shape-copying responses could be drawn from three different patients, and so on. By using this approach, we not only demonstrate many of the quirks found in responses within a neuropsychological context but also ensure that the sample to be rated includes both easy and difficult judgments, thereby avoiding bias in our rating quality in either direction.

Additionally, we provided all raters with both the BIT-c and NET manual instructions in English. For the copying and drawing subtests, instructions were provided in both English and German, as the two manuals differ substantially for these tasks. For the other subtests, we followed the German manual, which is the same as the English BIT-c instructions. All additional materials provided in the manual were made available to raters, such as stencils for measuring deviations of the bisection or for finding the targets in the letter-cancellation subtests, as well as a table to convert raw values into standard values (as is conducted in the NET).

#### 2.1.3. Procedure

Most raters came into our lab on two separate occasions. Due to scheduling issues, some of the expert raters completed the task in one session. We counterbalanced the order in which raters received the tests: (1) letter cancellation and line bisection, (2) figure and shape copying, and (3) representational drawing. Subtests were blocked. Thus, raters did not rate each patient at a time but rather one subtest at a time. This was in order to measure the time required for each subtest. The order in which the test results from different patients were presented was randomized but constrained by the requirement that repeat presentations of the same test results from the same patient were separated by at least two test results from different patients. This constraint was introduced to reduce the likelihood that raters would immediately recognize having seen that test result before. Each rater took between two and three hours.

The naïve group was split into two subgroups—with and without practice. Experts never received any practice. Practice for each subtest was always completed immediately before the experimental rating of the same subtest and consisted of three examples that we predicted to be uncontroversial. Raters gave their scores and reasoning, and the experimenter corrected any errors. Raters then scored ten patient files twice each. They were not informed about the repetition but were asked to rate each patient file separately in the instructions. However, it should be noted that repetitions are readily apparent for the copying/drawing subtests.

After completing each subtest, we asked the raters to fill out a questionnaire that inquired about any strategies used, as well as what they found easy and difficult when rating that particular subtest. This was in order to qualitatively analyze the ratings and the strategies employed. If the rated subtest was one of the copying/drawing subtests, participants were asked to copy or reproduce the relevant figures themselves after completing the questionnaire. Specifically, they were instructed to draw one version that would only receive a point for arrangement of details according to the NET manual, and another version that would just fail to receive a point for the arrangement criterion.

#### 2.1.4. Data Analysis

For the analysis of the NET, the raw values—that is, the direct performance outcomes obtained in each subtest (e.g., number of crossed-out targets, deviation from midline, scoring of the copies)—are converted into standardized values ranging from 0 to 10 according to the scoring table provided in the NET manual. These conversions differ between subtests to account for their specific scoring characteristics. For example, in the letter-cancellation subtest, a maximum of 40 targets can be crossed out. If a patient crosses out 0–1 target, the standardized score is 0; 2–3 targets correspond to a score of 0.5; and so on, in increments of 0.5, up to a maximum standardized score of 10 for 40 correctly identified targets. For the entire NET battery (conventional and behavioral part), each patient can then receive a maximum of 170 points. According to the manual [13], the following cut-off criteria should be used: (1) ≤72 points for severe neglect, (2) 73–135 points for strong neglect, (3) 136–166 points for mild neglect, and (4) ≥167 points for no neglect. Since we only had the capacity to rate our patients on a subset of subtests, we converted the cut-off values into percentages (our maximum standardized total score being 60 points): (1) ≤42.35% for severe neglect; (2) 42.36–79.41% for strong neglect; (3) 79.42–98.23% for mild neglect; and (4) ≥98.24% for no neglect. We then adjusted these thresholds to the maximum score achievable on our reduced version. Accordingly, the adjusted cut-offs are: (1) ≤25.41 points for severe neglect; (2) 25.42–47.65 points for strong neglect; (3) 47.66–58.59 points for mild neglect; and (4) ≥58.60 points for no neglect.

When setting a similar criterion for the BIT-c [11], it is important to consider that the different BIT-c subtests contribute unequally to the total score because their raw scores are not standardized. In the original BIT-c, this results in an incidental weighting. For instance, the letter cancellation (maximum 40 points) contributes much more heavily to the total score than the line bisection (maximum 9 points) or the copying/drawing subtests (1 point each). Since our adapted version includes only a subset of the original subtests, this imbalance would shift: the remaining copying/drawing subtests would be relatively over-represented when compared to their contribution in the full BIT-c. To preserve a weighting pattern similar to the original BIT-c, we explicitly assigned each subtest a weight corresponding to its proportional contribution to the total score in the subset used (subset weight = (maximum possible value of subtest)/(maximum total sum score of used subtests) × 100; letter cancellation: 75.47%; line bisection: 16.98%; copying subtests (star, cube, flower) and clock drawing: 1.89% each). This approach ensured that the relative influence of each subtest mirrored the original BIT-c’s incidental weighting as closely as possible.

The cut-off for detecting neglect was set at ≤ 28.09 points, which corresponds to the same proportional threshold (88.36%) as the original BIT cut-off (≤129 of 146 points), given that the maximum weighted score in our subset was 31.79 points.

### 2.2. Results

#### 2.2.1. Differences in Diagnoses Based on the Total Sum Score

**Figure 1:**
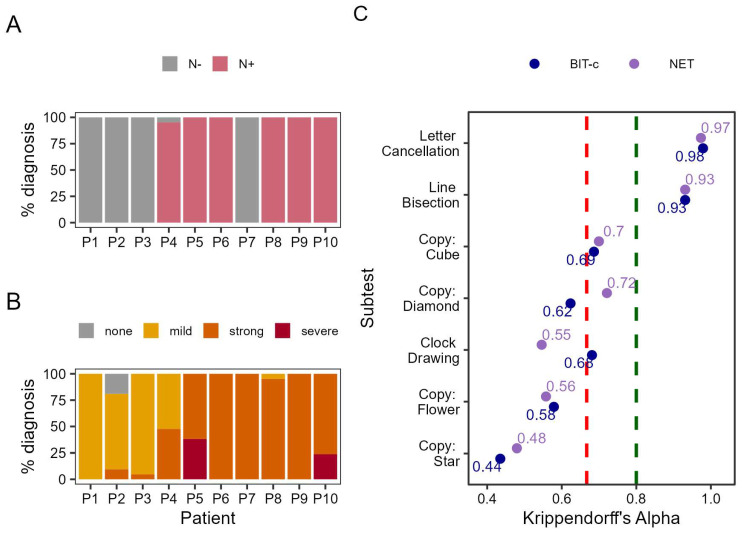
Percentage of raters assigning neglect diagnoses for each patient in (**A**) BIT-c and (**B**) NET and (**C**) consistency between raters per subtest. Note. Percentages reflect the proportion of the 21 raters assigning each diagnosis. Numbers on the *x*-axis refer to the patient ID. (**A**) Diagnoses based on the BIT-c criteria; note that for the BIT-c, only a cut-off for a general diagnosis is made. Grey bars indicate the absence of neglect (N−), while light red marks indicate neglect diagnoses (N+). (**B**) Meanwhile, the NET differentiates between presence and absence of neglect, as well as different severities as indicated by the colors grey (no neglect), orange (mild neglect), dark orange (strong neglect), and red (severe neglect). (**C**) Krippendorff’s alpha for each subtest and test battery (BIT-c in dark blue, NET in light purple). Dashed vertical lines indicate commonly used interpretation thresholds, with values above 0.67 (red dashed line) indicating agreement sufficient for tentative conclusions or “moderate agreement” and values above 0.80 (green dashed line) indicating “satisfactory agreement” [36,37].

First, we examined the level of diagnostic agreement among raters based on the total sum score (averaged between first and second rating) of the BIT-c [11]; i.e., whether each patient suffered from neglect or not according to the chosen cut-off score (≤28.09 points). As can be seen in Figure 1A, the agreement is very high for nine out of the ten patients—five of whom were labeled as neglect patients (N+) and four as non-neglect patients (N−). We found disagreement only in the evaluation of Patient 4. Here, 20 of the 21 raters assigned a diagnosis of neglect, while one did not. Overall agreement between raters for all 10 patients was notably high: 99.52%.

In the German NET [13], the diagnostic evaluation is more finely graded and distinguishes between no neglect, mild neglect, strong neglect, and severe neglect. As is best practice, we used the sum of the standard values (i.e., averaged across the first and second ratings) to assess neglect severity based on the raters’ evaluation. This graded structure naturally makes the NET more susceptible to variability in rater evaluations (see Figure 1B). Here, the agreement is perfect for four of the patients (Patients 1, 6, 7, and 9). One rater disagreed for Patient 3 (suggesting a more severe case of neglect) and another rater disagreed for Patient 8 (suggesting a less severe case of neglect). For another three patients, there was considerable disagreement in the severity of neglect (Patients 4, 5, and 10). Most notably, there was substantial variability in rating performance for Patient 2: 19.02% of raters rejected a diagnosis of neglect, 71.42% assigned a diagnosis of mild neglect, and 9.52% assigned a diagnosis of strong neglect. The overall agreement between raters was 85.24%.

These NET results contrast with the BIT-c evaluation, for which almost all raters agreed on their diagnosis. This is likely due to the use of (weighted) raw scores which overemphasize the cancellation subtests that are more objectively scored. In contrast, since the NET assigns equal weight to all subtests through standardization, it is more sensitive to subjective differences in evaluation. Interestingly, this also results in quite different diagnoses depending on whether the NET or BIT-c is used. According to the NET, nearly all patients would be classified as having neglect. However, only one patient was classified as not having neglect, and even then, only by 19.02% of raters—the majority assigned mild neglect. This suggests that the NET categorization is more sensitive to subtle differences in performance. To explore this further, we examined how results would change if mild neglect were reclassified as no neglect. Notably, the inter-rater agreement only improved to 87.14%, a gain of just 1.90 percentage points.

#### 2.2.2. Differences in Diagnoses Between First and Second Rating

We asked our raters to rate all of the patient files twice. For the BIT-c results, the overall agreement was 99.05%. Here, only two raters changed their rating in a way that affected the diagnosis—one rater became more lenient while the other became stricter. For the NET results, the overall agreement between first and second rating was 90.48%. Eleven out of the 21 raters changed their scores enough to affect the diagnosis. No clear pattern of raters becoming more lenient or strict was detectable. When comparing the two batteries, we also found that overall agreement was higher for the BIT-c. Several factors could explain this—the BIT-c could, of course, simply have an inherently higher consistency, but the difference may also reflect the incidental overweighting of the more objective cancellation subtests and the use of a binary criterion for assessing the copying and drawing subtests. The binary criterion could reduce the chance of arriving at a different rating for the same copy (BIT-c with scores of 0 or 1 points vs. NET with scores of 0, 1, 2, or 3 points). Additionally, the BIT-c binary rating could be completed much more quickly, meaning that raters could encounter the same drawing for the second time much sooner than during the NET copying/drawing evaluation. Since the NET might be overly responsive to signs of neglect, we applied a stricter criterion by collapsing mild neglect to the no neglect category. Again, this only led to an increase in the NET agreement between the first and second ratings across all raters by 1.90 percentage points.

#### 2.2.3. Agreement Between Raters on Different Subtests

The total sum score is based on the weighted sum of individual subtests. The subtests differ considerably in aspects that influence the rating and the difficulty of consistent ratings. Therefore, we investigated differences in agreement between raters for each subtest separately. Unlike the sum score, the subtests work on different scale levels: the copying/drawing subtests are binary, the line bisection subtest is ordinal, and the letter cancellation is on the ratio scale. To account for this, Krippendorff’s alpha was computed for all subtests to allow comparisons across different ratings scales and between the test batteries. This approach assumes only ordered relationships between scale points, ensuring consistency across rating types. Generally, Krippendorff’s alpha should be greater than 0.67 to indicate moderate agreement, while values above 0.80 are considered to reflect satisfactory agreement [36,37].

We calculated Krippendorff’s alpha for each selected subtest in the BIT-c and found that they ranged from α = 0.44 (for star copying) to α = 0.98 (for letter cancellation). As can be seen in Figure 1C, the copying/drawing subtests ranged from α = 0.44 for the star copying to α = 0.69 for the cube copying, placing them either in the unacceptable range or just at the threshold of moderate agreement. This further supports the observation that the copying and drawing subtests are highly subjective, while subtests like letter cancellation (α = 0.98) and line bisection (α = 0.93) show excellent consistency, robustly higher than the satisfactory threshold, likely due to their more objective criteria.

We calculated the same parameter for the NET and found comparable results for the letter-cancellation (α = 0.97) and line-bisection (α = 0.93) subtests. For the copying subtest, the results seemed mostly comparable as well—sometimes the NET showed higher consistency (i.e., in the star copying), while sometimes the BIT-c did (i.e., in the flower copying). Only in the diamond copying did we find substantially better consistency for the NET. However, the diamond copying task is not part of the original BIT-c. Considering these results, the more elaborate evaluation criteria of the NET did not improve consistency.

The manual of the BIT-c states cut-off values for each subtest. Therefore, we calculated how often a disagreement in subtest scoring would affect the diagnosis if that diagnosis were based just on the letter-cancellation or the line-bisection subtests. Despite the high agreement of raters for those two subtests (Figure 1C), we found that even this mild disagreement could lead to variability in diagnosis for some patients: For letter cancellation, the scoring disagreement resulted in different diagnoses for Patients 6 and 9. For both patients, 4.76% of raters assigned a non-neglect diagnosis while 95.24% assigned neglect. For the line bisection subtest, we also found disagreement for two patients (Patients 8 and 10). For Patient 8, 14.29% of the raters gave the patient a score that would warrant a neglect label, while 85.71% rated them as non-neglect. For Patient 10, 23.81% of raters deemed the patient to be a neglect patient based on the line bisection subtest, while 76.19% deemed the patient to belong to the non-neglect group.

#### 2.2.4. Differences Between First and Second Ratings on Different Subtests

Next, we examined the agreement between the first and second ratings for each of the different subtests. To do so, we calculated a repeated measures correlation for each subtest. Most correlation coefficients were above 0.9 (all Bonferroni-corrected *p*-values < 0.001; see supplemental Table S1 for the BIT-c and Table S2 for the NET), indicating strong agreement between the first and second ratings. In three cases, the coefficient fell below 0.9: the BIT-c copying star with r = 0.85, the NET copying star with r = 0.85, and the NET copying diamond with r = 0.88.

Again, we examined how applying individual subtest cut-off criteria affects the resulting diagnosis. For letter cancellation, we observed a change in Patients 2, 4, and 7. In all cases, raters became more sensitive to signs of neglect, and more patients received scores implying neglect on the second assessment. In most cases, this was a change in one rater (Patients 2 and 4). For Patient 7, two raters changed their scores so substantially that they fell below the cut-off for letter cancellation. Interestingly, in the line bisection subtest, the raters tended to apply the criteria for neglect less strictly, and we observed changes in Patients 8 and 10. For Patient 8, one rater’s revised score moved the patient above the neglect threshold; for Patient 10, three raters’ revised scores did the same.

#### 2.2.5. Differences Between Rater Groups

Our 21 raters fell into three groups—neuropsychological experts with clinical experience, naïve participants with practice, and naïve participants without practice. To examine the influence of experience on rating performance, we calculated the agreement of the diagnosis based on the weighted total score per group and battery. In line with our previous overall agreement calculations, agreement on the BIT-c was generally higher than on the NET. Furthermore, we found that the experts showed equal or slightly higher agreement compared to non-experts (agreement values: for the BIT-c, 98.57% for naïve without practice, and 100% for both naïve with practice and experts; for the NET, 88.57% for naïve without practice, 85.71% for naïve with practice, and 91.43% for experts).

Next, we examined the agreement between the first and second ratings as a function of expertise by calculating repeated measures correlations for each group. The general agreement between the first and second ratings was high (i.e., all correlation coefficients were at least 0.94). For the BIT-c, we found the following repeated measures correlations: naïve without practice: r = 0.98, *p* < 0.001, CI = [0.97, 0.99]; naïve with practice: r = 1.00, *p* < 0.001, CI = [1.00, 1.00]; and experts: r = 1.00, *p* < 0.001, CI = [1.00, 1.00]. Similarly, the agreement between the first and second ratings was also high for the NET in all groups: naïve without practice: r = 0.94, *p* < 0.001, CI = [0.91, 0.97]; naïve with practice: r = 0.96, *p* < 0.001, CI = [0.96, 0.98]; and experts: r = 0.99, *p* < 0.001, CI = [0.99, 1.00]. This demonstrates that consistency across different ratings by the same raters is generally very high, particularly among experts.

#### 2.2.6. Self-Rated vs. Actual Consistency

Overall, the actual consistency of all raters was high for both the BIT-c and NET. The self-rated consistency, assessed after rating all patients on a given subtest, also tended to be high at around 4.00 to 4.75 (BIT-c: M = 4.24, SD = 0.49; NET: M = 4.17, SD = 0.57) on a scale from 1.00 to 5.00. However, some raters showed low self-rated consistency (e.g., one naïve rater had an average rating of 3.00 (SD = 1.15) for the BIT-c and 2.50 (SD = 1.00) for the NET). We calculated Cohen’s kappa per rater between first and second rating, as a measure of actual consistency. Generally, higher actual consistency was paired with higher self-rated consistency as expected. However, there were three (out of 21) notable exceptions: one naïve rater had a very high actual consistency for the NET (kappa = 0.95) but the lowest self-rated score (M = 2.50, SD = 1.00); another naïve rater had high consistency for both the BIT-c (kappa = 0.98) and the NET (kappa = 0.96) but a relatively low self-rated score (for the BIT-c: M = 3.67, SD = 0.58; for the NET: M = 3.5, SD = 1.00); and one expert rater had an almost perfect actual consistency of kappa = 0.99 for the NET but only a modest self-rating (M = 3.75, SD = 1.26).

#### 2.2.7. Efficiency

**Figure 2:**
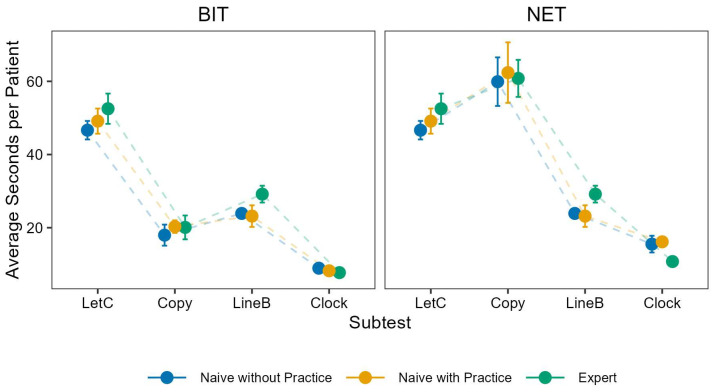
Average rating times per patient for each subtest in the BIT-c and NET. Note: Error bars represent the standard error of the mean. The times for the figure-and-shape-copying tasks include ratings of four drawings (star, cube, flower, and diamond).

To assess the time required to evaluate patient files across subtests, we calculated the mean time per patient (see Figure 2). Irrespective of rater group, some subtests took longer than others. The longest subtests to evaluate were letter cancellation (M = 49.73 s; SD = 8.28 s) and the NET figure-and-shape-copying evaluation (M = 61.88 s; SD = 16.43 s). The quickest were the BIT-c figure-and-shape-copying evaluation (M = 19.67 s; SD = 6.34 s) and representational drawing (BIT-c: M = 8.45 s; SD = 2.64 s; NET: M = 14.74 s; SD = 4.73 s). Time required to assess a given subtest was quite similar across the three groups. In fact, experts took slightly longer than non-experts. While individual durations may appear short, the cumulative time across subtests and patients was substantial.

### 2.3. Interim Discussion

We investigated the consistency of the BIT-c and NET evaluations both between and within raters of varying levels of expertise. Note that the data reflect only the rating component of the diagnostic process, not the administration of the tests by each rater. Additionally, because the sample was not randomly selected, we do not present this as a traditional reliability study. Overall, we found high inter- and intra-rater agreement. Nevertheless, the diagnosis did change between raters and between ratings by the same rater. This indicates that even under controlled conditions, some degree of variability cannot be avoided. Investigating the subtests separately revealed the copying and drawing subtests to be the primary drivers of rating variability. Their dependence on more subjective criteria constitutes a structural weakness. Notably, using more elaborate criteria, as done in the NET, did not seem to improve consistency much, if at all. This suggests that increasing the formal complexity of instructions alone does not necessarily translate into more consistent ratings. Moreover, the more elaborate criteria came at a cost: assessment time per patient increased substantially. The lack of consistency in the copying/drawing subtests influenced consistency for the BIT-c less than for the NET. This is likely due to the NET placing greater weight on the figure-and-shape-copying subtest, whereas the influence of these subtests on the BIT-c is minimal. Interestingly, there was no strong difference between the three groups (experts, naïve raters without practice, and naïve raters with practice). This raises questions about whether clinical experience alone can sufficiently mitigate rating inconsistencies. Despite the high actual consistencies, some raters from all groups perceived themselves as less consistent, which may reflect uncertainty in their own judgment, despite actually performing consistently. Confidence also varied across raters and subtests.

While the consistency was high, it should be stressed that these evaluations took place under ideal circumstances. No time pressure was imposed on raters, and all documents had been sorted with relevant information highlighted (e.g., the column for the conversion of raw to standard values for the NET). This is in stark contrast to real-life applications. Consequently, the consistency estimates observed in the present study likely represent an upper bound that may not fully generalize to routine clinical settings. In clinical settings, raters are often under significant time pressure, with fewer opportunities for supervision or second opinions. Research suggests that this has meaningful consequences: one study of residents in internal medicine found that diagnostic error was 37% higher under time pressure [9], and another showed that time pressure not only increased error rates but also decreased raters’ confidence in their judgments [38]. In a research context, time pressure may be lower, but as diagnoses are typically not established under double-blind conditions there remains a potential risk of expectancy or confirmation bias.

To address some of the issues with evaluating neglect tests, we will introduce a different approach in the second part of our paper: a computer vision-based automation tool for the evaluation of a subset of neglect tests found in the BIT-c and NET.

## 3. Part 2: Utilizing Computer Vision for Reliable Evaluations of the BIT-c

With the rapid growth of artificial intelligence (AI) in the early 2020s, AI-based approaches in diagnostics have proliferated. This trend has extended to neuropsychological diagnostics. For example, Langer and colleagues [39] used deep learning methods to automate the evaluation of the Rey–Osterrieth Complex Figure Test—a test used to evaluate deficits in memory and visuoconstruction. Their program automated the entire evaluation process and reached superior accuracy compared to online raters as well as clinicians [39]. Similarly, AI has been used to analyze speech and language patterns in patients with traumatic brain injury [40]. Further examples include automating the evaluation of the clock drawing test for dementia screening [41] and differentiating phenotypes of progressive supranuclear palsy [42].

Here, we present a computer vision (CV)-based approach to support the evaluation of the BIT-c and NET—the Computer-based Analysis of Neglect Deficits On paper (CANDO). CV is a subfield of AI concerned with enabling machines to interpret and extract meaningful information from visual inputs. Its main goal is the detection, identification, and analysis of objects or patterns in images or videos. This can be achieved through manually developed algorithms (classical or traditional CV) or through deep learning approaches [43,44]. By automating the evaluation, we aimed to increase efficiency and reduce the potential biases in manual evaluation.

We opted for a classical CV pipeline rather than a machine learning (ML) approach for several reasons. First, CANDO is intended to supplement rather than replace expert evaluation, a role for which transparency, auditability, and perfect test–retest reliability are particularly valuable—properties that classical CV provides by construction. Second, when CANDO produces an incorrect score, the failure mode is interpretable and traceable to a specific rule, which is much harder to achieve with deep learning approaches. Conceptually, CANDO already incorporates elements similar to archetypal comparison approaches by constructing idealized geometric representations of patient drawings and evaluating deviations from these structures. However, unlike learning-based visual similarity approaches, these deviations are quantified through explicit geometric criteria. An additional advantage of this deterministic approach is that the evaluation criteria can be explicitly adjusted depending on the intended application. For example, researchers aiming to define highly specific control groups may choose stricter geometric thresholds, whereas studies focusing on mild neglect symptoms may prefer more permissive criteria in order to maximize sensitivity. Third, while ML-based scoring has been successfully applied to adjacent neuropsychological tests (e.g., the Rey–Osterrieth Complex Figure [39] and clock drawing [41]), these efforts relied on substantially larger annotated datasets than are currently available for the BIT-c/NET. Moreover, generating such datasets is particularly challenging in the context of neglect assessments because, as demonstrated in Part 1, several BIT-c/NET evaluation criteria are themselves underspecified and associated with considerable inter-rater variability. Consequently, constructing sufficiently large and consistent expert-annotated training datasets would require substantial additional standardization efforts. While ML-based approaches may ultimately prove advantageous for highly variable or representational drawing tasks, they are currently difficult to implement robustly within the BIT-c/NET framework. Finally, our deterministic pipeline runs on a standard consumer-grade laptop and requires no dedicated GPU hardware, which lowers the barrier to clinical deployment. We see CANDO as establishing a rule-based baseline against which future ML approaches to BIT-c/NET scoring can be benchmarked.

To assess accuracy and validity, we systematically compared the automated results against ground-truth values. This ground truth was derived from manual evaluations by one of the authors (LB). When discrepancies arose between the CV and manual evaluations, we determined which was incorrect. Manual errors were corrected, while CV errors were not. Thus, all errors reported in the following analysis reflect failures of CANDO. Accordingly, we aimed to evaluate whether CANDO can achieve high agreement with manually established ground-truth evaluations while improving the objectivity, reproducibility, and efficiency of scoring across different BIT-c/NET subtests. We expect high accuracy from our automation across the different BIT-c/NET subtests and, by extension, a robust, objective alternative to traditional manual evaluation.

### 3.1. Materials and Methods

#### 3.1.1. Sample Description

The diagnostic data used in this study were collected prior to the development of the automation system and are derived from three different studies. The patient data therefore stem from a retrospective convenience sample. The scans originated from assessments conducted across various hospitals, rehabilitation centers, and the neuropsychological outpatient clinic of Ludwig-Maximilians-Universität München, while a smaller number of patients participated independently for research purposes. While this ensures very “natural” scans, it also introduces variability in the data: different patients completed different subsets of subtests, and some were assessed using the BIT-c (e.g., the copying task with the Necker/wireframe cube) while others used the NET adaptation (e.g., the copying task with the diamond). This also resulted in a larger proportion of patients having right-hemisphere brain damage compared to left-hemisphere brain damage, with varying presence of neglect and visual field defects (VFD). All patients who completed at least one of the BIT-c/NET subtests in accordance with the scan selection criteria are characterized below. A detailed description of the subsamples for each subtest is provided in Table S3 of the Supplemental Material.

Test sheets were excluded if (1) markings were produced with very low contrast writing tools (e.g., thin pencil markings or red-colored pens); (2) the patient was unable to comply with test instructions; (3) the print of the test sheet was distorted or cut off; (4) clinicians added markings to indicate initially missed targets; (5) drawings in the figure-and-shape-copying subtest extended substantially beyond the designated drawing area; or (6) extensive corrections were made on the test sheet, e.g., crossing out one drawing and reproducing another next to it in the figure-and-shape-copying subtest or marking multiple midpoints in the line bisection subtest. These criteria were chosen because such conditions can introduce ambiguities that complicate reliable automated analysis and, in some cases, would also substantially affect manual evaluation. For example, low-contrast markings may become difficult to distinguish from the background during grayscale conversion and are also more likely to be overlooked during manual scoring. Distorted scans can interfere with geometric normalization procedures but would also complicate the use of template scoring sheets for manual scoring, and extensive corrections or clinician-added marking make it difficult to determine which markings should be attributed to the patient. Less severe deviations from these criteria (e.g., crossing out only part of a figure rather than the entire figure) were retained in the dataset and, where they resulted in errors, are discussed in the subtest-specific error analyses. As such cases should be avoided following the best-practice guidelines (see Section 4.3), they are not expected to occur during routing future use of CANDO.

In total, we had usable diagnostic scans from 101 patients (46 females and 55 males). The patients were on average M = 63.25 years old (SD = 14.80 years). The youngest patient was 21 years old, and the oldest was 87 years old. Most patients had right-sided brain damage, and more than half of them had neglect, while a quarter suffered from VFD (see Table 2), according to their medical report. Importantly, our sample is not a random or representative sample and, thus, no conclusions on the prevalence of neglect, VFD, or their co-occurrence can be drawn from the values found in our sample.

#### 3.1.2. Data Preparation

Sometimes information regarding the test date, pseudonym, or other patient details was written on the front side of the scans (instead of the back). We removed this information from the scans (using the software “Microsoft Photos” (Windows 11 app) ) prior to running them through the automation pipeline. The NET version of the line bisection subtest does not include a fiducial marker; an arrow was therefore added to indicate the bottom of the page.

#### 3.1.3. Technical Implementation: Overview

**Figure 3:**
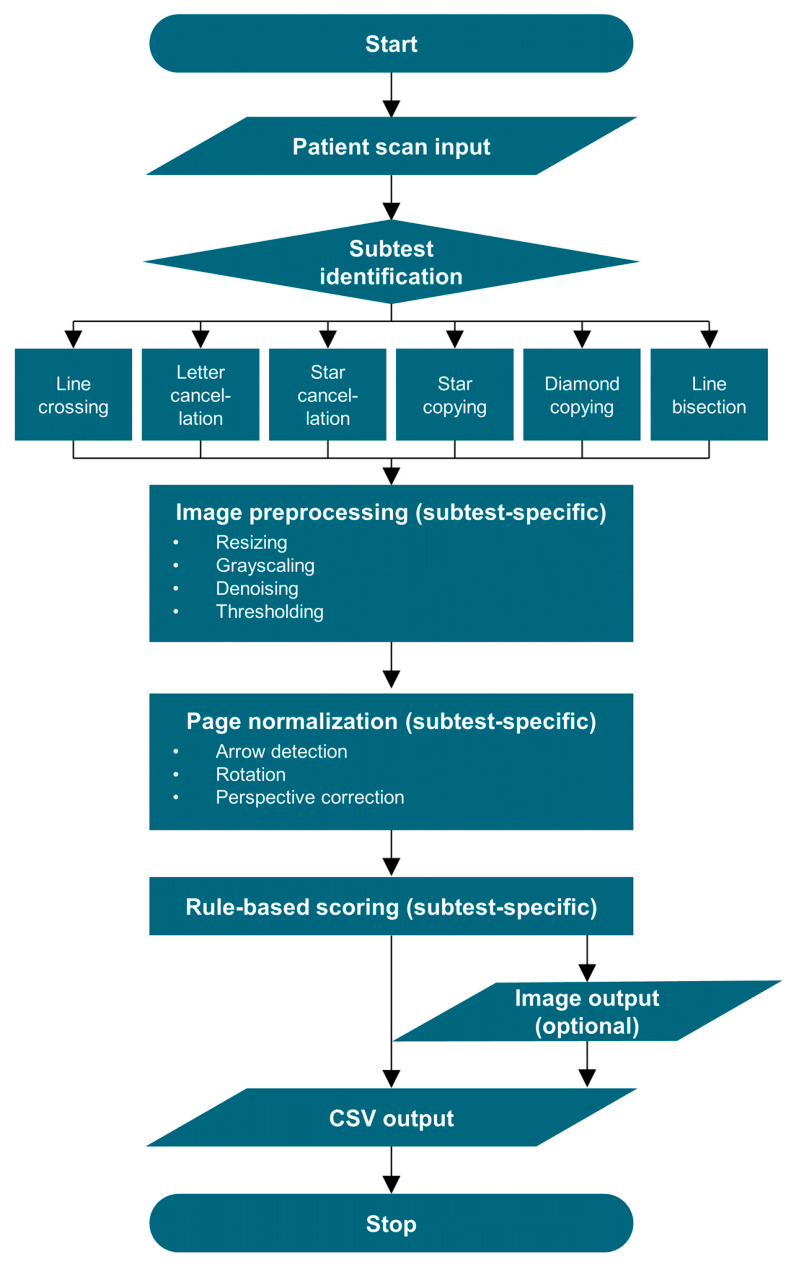
Simplified overview of the general CANDO workflow from scanned test sheet acquisition to automated extraction of subtest-specific outcome variables and output of a structured data file (CSV format). Individual subtests additionally tailored contour-, corner-, or line-based analyses depending on task requirements.

All six subtests were analyzed using a custom CV-based pipeline implemented in Python (version 3.11.5) and OpenCV (version 4.8.1.78). CANDO applies deterministic computer vision techniques to replicate the scoring rules of BIT-c and NET in a standardized and reproducible manner. Unlike machine learning-based approaches, this approach does not rely on training data but instead operationalizes predefined scoring criteria explicitly, thereby emphasizing transparency, interpretability, and reproducibility of the evaluation process. CANDO accepts digital images of completed test sheets and outputs a structured CSV file containing subtest-specific outcome variables reflecting target detection, bisection behavior, and drawing characteristics. The pipeline can also save copies of evaluated test sheets and supports single-file analysis (one patient, one subtest). Across all subtests, images underwent a similar set of preprocessing steps, including standardization of image resolution, noise reduction, and edge-based feature enhancement to facilitate subsequent detection and segmentation steps (see Figure 3). Each subtest applied a tailored sequence of contour-, corner-, or line-based analyses to normalize image orientation, isolate subtest-relevant regions, and extract automated measures of interest, e.g., the number of crossed-out targets, bisection scores, or completeness scores. The complete source code and usage instructions are available at https://osf.io/qdfk9. Use of CANDO requires lawful access to the official test materials of the BIT or the NET. An overview of all automated output variables is provided in Table S4 in Supplementary Section S3, and a detailed technical description of the implementation is provided in Supplementary Section S4. Here, we will focus on the output from each subtest.

#### 3.1.4. Technical Implementation: Line Crossing

In the line-crossing subtest, patients are asked to cross out small lines spread across the test sheet. For evaluation, the number of lines the patient crossed out is counted. The program produces a report that includes the conventional total score (CSV column name: *LineC*), the left/right counts (*LineC_LS, LineC_RS*) and the NET standard value (*LineC*_SV).

**Figure 4:**
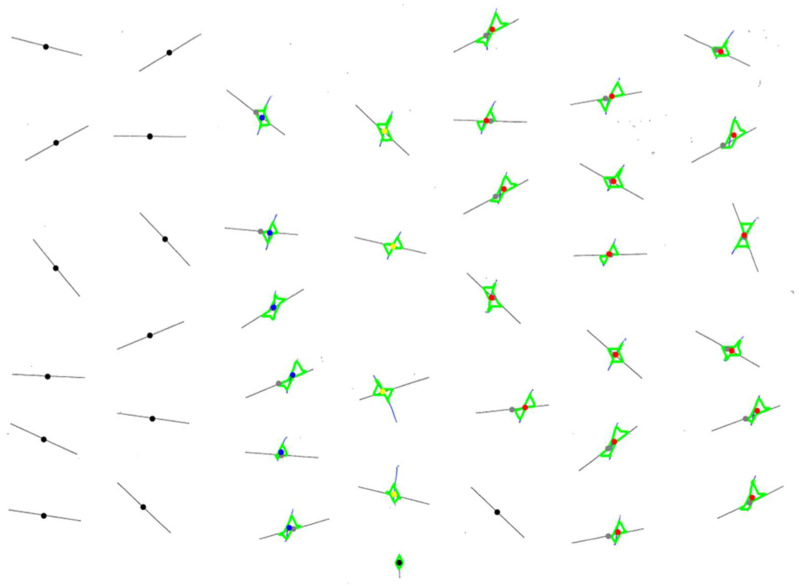
Example of a processed line-crossing subtest. Note: processed image of the line-crossing subtest for a neglect patient. Patients are asked to cross out all small lines on the page. In accordance with the instructions, the lines in the middle of the test sheet (marked by yellow dots) are ignored for the evaluation and scoring. Blue and red dots identify targets on the left and right sides of the test sheet that were correctly crossed out, respectively. Each target line is marked with a dot at its true midpoint—black if no crossing was detected, grey for a detected crossing.

If the line-crossing subtest is evaluated directly via the *line_crossing.py* file, the program returns a tuple listing these values: *(LineC_LS, LineC_RS, LineC, LineC_SV)*. Furthermore, if the final step (*scoring_img*) is visualized, it returns the evaluated version of the respective test sheet, e.g., Figure 4. In the visualized output, each target is marked with a dot at its midpoint—black if no marking by the patient was detected, grey if it is. All markings made by the patient are categorized as left-sided (in blue), right-sided (in red), or middle (in yellow). Markings in the middle are ignored for evaluation, as instructed in both the BIT-c and NET manuals.

#### 3.1.5. Technical Implementation: Letter Cancellation

In the letter-cancellation subtest, patients are asked to cross out all letters E and R among distractor letters. The score corresponds to the total number of Es and Rs crossed out (CSV column name: *LetC*). In addition to the conventional total score, our pipeline reports separate left/right counts (*LetC_LS, LetC_RS*) and NET standard value (*LetC_SV*).

**Figure 5:**
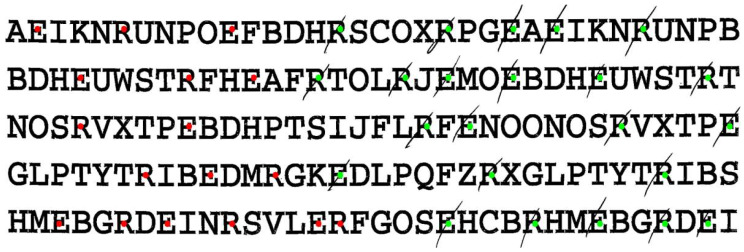
Example of a processed letter-cancellation subtest. Note: processed image of the letter cancellation subtest for a neglect patient. Patients are asked to cross out all Es and Rs on the page. Green dots indicate targets detected as crossed out, while red dots indicate those that were not.

If this subtest is evaluated directly via the *letter_cancellation.py* file, the program similarly returns a tuple listing these values for a given patient: *(LetC_LS, LetC_RS, LetC, LetC_SV)*. Additionally, the scored test sheet can be visualized as shown in Figure 5. Here, green dots indicate targets detected as crossed out, while red dots indicate those that were not.

#### 3.1.6. Technical Implementation: Star Cancellation

In the star-cancellation subtest, patients are asked to cross out all small stars among larger stars, distractor letters, and distractor words. Similarly to the previous two subtests, the score is based on the total number of small stars crossed out (CSV column name: *StarC*). Additionally, the program reports separate left- and right-side counts (*StarC_LS, StarC_RS*) and the NET standard value (*StarC_SV*).

**Figure 6:**
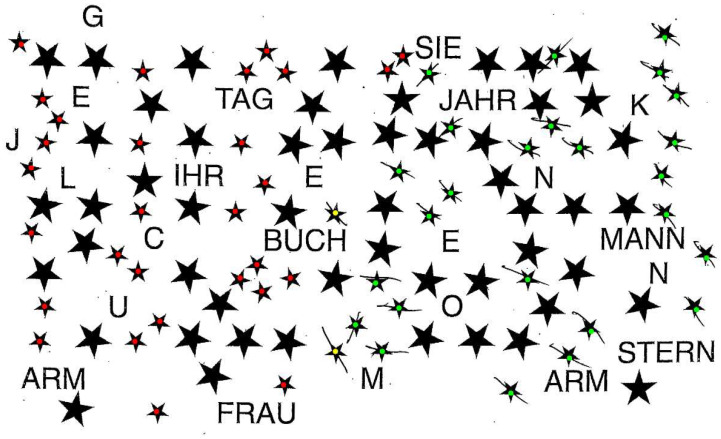
Example of a processed star-cancellation subtest. Note: processed image of the star-cancellation subtest for a neglect patient. Patients are asked to cross out all small stars on the page. As instructed in the manuals, the two small stars in the center of the test sheet (marked by yellow dots) are excluded from scoring. Green dots indicate targets that were detected as crossed out; red dots indicate those that were not.

As with the previous two subtests, a patient can also be evaluated directly in the *star_cancellation.py* file. Running this script returns a tuple with all relevant values: *StarC_LS, StarC_RS, StarC, StarC_SV*. An example visualization is shown in Figure 6, where green dots indicate the target stars detected as crossed out, while red dots indicate those that were not. The two small stars in the center, typically crossed out by the clinician as examples of the task, are excluded from scoring and are marked in yellow.

#### 3.1.7. Technical Implementation: Copying Star

In the figure-and-shape-copying subtest, patients are asked to copy a set of figures. Here, we describe the evaluation of the four-pointed star. Under the NET criteria, one point is assigned for each of three attributes: complete shape (CSV column name: *CopyStar_S*), presence of all details (*CopyStar_D*), and correct arrangement (*CopyStar_A*). The final score is then the sum across these three attributes (*CopyStar_NET*), converted to a standard value (*CopyStar_SV*). Under the BIT-c criteria, the drawing is simply evaluated for completeness and assigned a binary score of 0 or 1 (CSV column name: *CopyStar*). A point is awarded if the drawing received points for both shape and details, indicating a closed shape with the presence of all details (i.e., all four points).

Scoring proceeds in three parts. First, the shape score evaluates whether the main edges and overall radial geometry are preserved. This requires all expected edges to be detected and the outer and inner corner to show a relatively consistent radial structure around the center of the figure (maximum radial deviation ≤ 100 pixels). Second, the detail score requires the shape score to pass and verifies the presence of all expected outer and inner corners, indicating a complete four-pointed star (exactly eight corners required). Third, the arrangement score evaluates symmetry and spatial organization, including the alignment and relative geometry of the star’s corners and inner structure. This includes comparable horizontal and vertical spans (difference ≤ 200 pixels), sufficient alignment of opposing tips (maximum misalignment ≤ 200 pixels), relatively uniform distances between intermediate corners (maximum distance deviation ≤ 100 pixels), and approximately correct internal angles (maximum angular deviation ≤ 40°, with at least two angles deviating by no more than 30° from the expected 110° angle).

**Figure 7:**
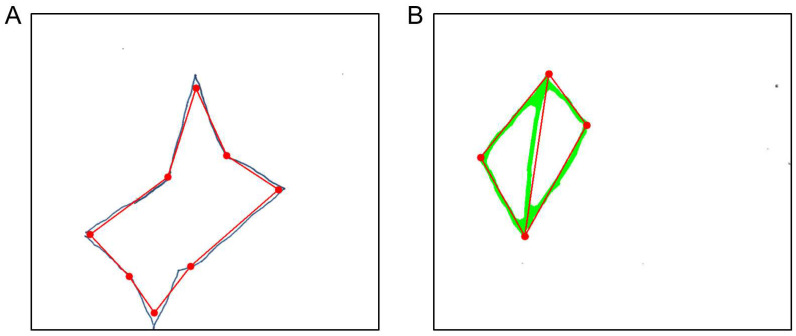
Example of a processed (**A**) star and (**B**) diamond from the figure-and-shape-copying subtest. Note. (**A**) Processed image of the star copying for a neglect patient. Patients are asked to copy a simple figure from a template presented on the left. Red dots show the detected corners of the patient’s drawing, while the red lines reflect the idealized star derived from these corners. This is overlaid on top of the patient’s drawing. (**B**) Processed image of the diamond copying for a neglect patient; this task is part of the German adaptation only. Red dots show the detected corners of the patient’s drawing, while the red lines reflect the idealized diamond derived from these corners. This is overlaid on top of the patient’s drawing, which is shown in green.

The star-copying subtest can also be evaluated directly via the *star_copying.py* file, which returns a tuple containing all relevant values: *(CopyStar_S, CopyStar_D, CopyStar_A, CopyStar_NET, CopyStar, CopyStar_SV)*. Furthermore, the patient’s drawing can be visualized, as shown in Figure 7A. In this figure, a red, idealized version of the star or archetype—derived from the edges and corners of the patient’s drawing—is overlaid on the original and compared against it for relevant features, i.e., the number of points and correct angles.

#### 3.1.8. Technical Implementation: Copying Diamond

The next component of the figure-and-shape-copying subtest involves copying a diamond with a middle line, which is part of the NET only. Scoring follows the same three-attribute structure as the star: shape (CSV column name: *CopyDiamond_S*), details (*CopyDiamond_D*), and arrangement (*CopyDiamond_A*), summed to produce a final score (*CopyDiamond_NET*) and converted to a standard value (*CopyDiamond_SV*). A hierarchical variant is also computed (*CopyDiamond_NET_hierarchical*), in which detail points require a shape point, and arrangement points require both shape and detail points. Although the BIT-c does not include this drawing, we applied its completeness criteria analogously—requiring a closed shape and presence of all details—and report it as *CopyDiamond*.

Here, scoring also proceeds in three stages. First, the shape score assesses whether all four outer edges of the diamond are present and form a closed figure (all four outer edges required). Second, the detail score shows whether the vertical bisector is present (vertical center line required). Third, the arrangement score assesses whether the corner angles are consistent with the expected diamond geometry, with deviations beyond a predefined tolerance treated as incorrect arrangement (ideally the angles between the vertical line and the outer lines of the diamond are 45° and the tolerance is ±7°). The arrangement score is zero if at least two of the four angles exceed this tolerance.

As with the star, the drawing can also be evaluated for a single participant in the *diamond_copying.py* file, returning the following tuple: *(CopyDiamond_S, CopyDiamond_D, CopyDiamond_A, CopyDiamond_NET, CopyDiamond, CopyDiamond_SV, CopyD_NET_hierarchical)*. An example visualization of an evaluated drawing is shown in Figure 7B, where the original patient drawing appears in green with the idealized diamond overlaid in red. This idealized version is then checked for closure of the drawing, presence of a midline, and correct arrangement of angles.

#### 3.1.9. Technical Implementation: Line Bisection

In this subtest, patients are asked to mark the midpoint of three horizontal lines. For scoring, the template is placed on top of the test sheet, indicating whether each marking should receive 3, 2, 1, or 0 points. The total subtest score is the sum across the three lines (CSV column: *LineB*), and for the NET, a standard value is also provided (*LineB_SV*). Additionally, we report the score for each line individually (top—*LineB_T*, middle—*LineB_M*, bottom—*LineB_B*). Each line score is accompanied by a direction indicator: ‘L’ if the marking fell to the left of center, or ‘R’ if to the right.

**Figure 8:**
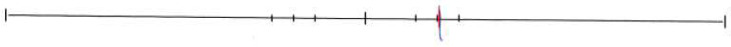
Example of a processed line bisection subtest. Note. Processed image of one line of the line bisection subtest for a neglect patient. Patients are asked to mark the midpoint of each of three horizontal lines, indicated here by the blue pen mark. The endpoints, actual midpoint, and point scale are marked in black. The detected bisection point is marked in red.

The line bisection subtest can also be evaluated directly via the *line_bisection.py* file, which returns the following tuple containing the values described above: *(LineB_T, LineB_M, LineB_B, LineB, LineB_SV)*. An example visualization is shown in Figure 8. The visualization overlays template-like markings indicating the point values for each zone, with the detected bisection point shown in red.

### 3.2. Results

#### 3.2.1. Line Crossing

**Figure 9:**
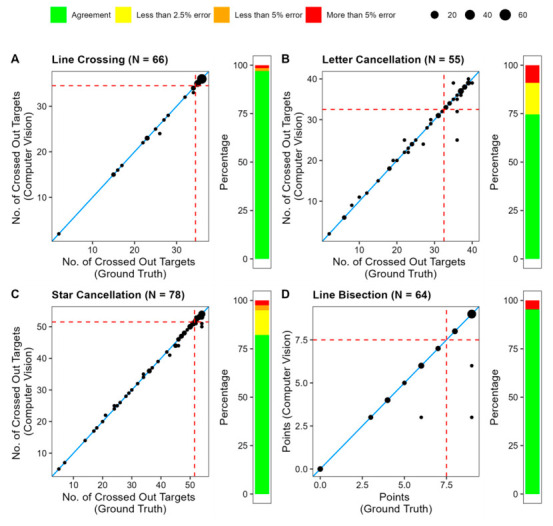
Agreement between CV detection and ground truth for (**A**) line crossing, (**B**) letter cancellation, (**C**) star cancellation, and (**D**) line bisection. Note. Each panel plots CANDO output against ground truth. Dot size reflects the number of overlapping cases at that coordinate. The color bar to the right of each plot presents the level of agreement between CANDO and ground truth. Four levels are used: green—full agreement; yellow—0% < disagreement ≤ 2.5%; orange—2.5% < disagreement ≤ 5%; red—disagreement > 5%. Bar height indicates the proportion of cases in each category. The blue diagonal represents the line of identity (i.e., perfect agreement). The red dashed lines indicate the values at which the diagnosis would change from non-neglect to neglect (i.e., cut-off values). Thus, data points that fall below the red horizontal line and to the right of the red vertical line represent cases that would have been identified as “normal” by ground truth but misdiagnosed as a neglect patient by CANDO. Such false positives occurred rarely, in three of the four subtests. Conversely, data points that fall above the red horizontal line and to the left of the red vertical line represent cases in which a neglect patient identified by ground truth was missed by CANDO. No such false negatives were observed.

First, we focus on the cancellation tests. For the line-crossing subtest, we had a sample of 66 brain-damaged patients with and without neglect. The average number of crossed-out targets detected by CANDO was M = 32.47 (Mdn = 36; SD = 6.95) compared to the ground truth of M = 32.52 (Mdn = 36; SD = 6.92). According to a Kendall tau correlation between the ground truth and CV count (see Figure 9A), the two measures correlate highly, τ = 0.998, *p* < 0.001. Agreement between the manually established ground truth and CANDO was likewise extremely high (ICC(A,1) = 0.999, 95% CI [0.999, 1.000]). Exact agreement was observed in 96.97% of cases, and 98.48% of evaluations differed by no more than one target.

Overall, CANDO was incorrect in the evaluation of two cases, failing to pick up one or two markings in each. In one case, the false negatives were caused by multiple crossings/zigzag crossing within the same target. In the other case, one marking was not detected, as it was very long, angled such that it was almost parallel to the target, and was drawn at the edge of the target—while CANDO can handle these problematic features individually, it struggles when all of them are present within the same target marking.

#### 3.2.2. Letter Cancellation

After applying the scan inclusion criteria, 55 patients could be included in our analysis. On average, the number of detected crossed-out targets was similar for CANDO and ground truth: M = 28.75 (Mdn = 32; SD = 9.95) for CANDO compared to M = 28.93 (Mdn = 33; SD = 10.08) for ground truth. The number of crossed-out targets as counted by CANDO correlated highly with those counted by the expert rater: Kendall tau τ = 0.927 (*p* < 0.001). Out of the 55 processed scans, CANDO made errors in 14 of them (Figure 9B).

Agreement between the manually established ground truth and CANDO remained very high (ICC(A,1) = 0.984, 95% CI [0.972, 0.990]). Exact agreement was observed in 74.55% of cases, and 90.91% of evaluations differed by no more than one target. The mean absolute difference was 0.62 targets.

**Figure 10:**
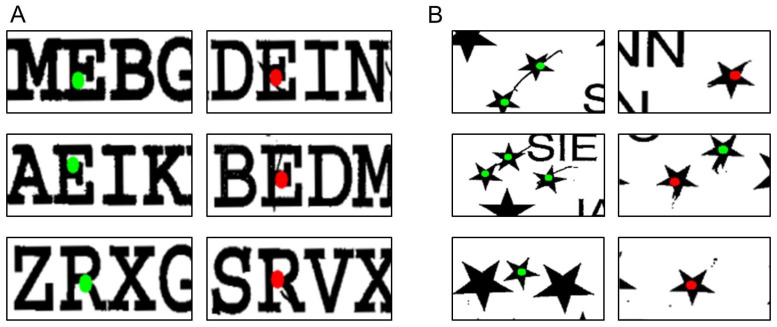
Examples of errors in the (**A**) letter- and (**B**) star-cancellation subtests. Note: (**A**) patients were asked to cross out all Es and Rs on the page. The left side depicts examples of letters incorrectly detected as crossed out. The right side depicts examples of letters incorrectly detected as not crossed out, despite being correctly marked by patients. A green dot indicates that a target letter was detected as crossed out, while a red dot indicates that it was not detected as crossed out. False detections can result from low-quality prints in which the target letter is very close to the distractors (see top-left example). False misses frequently occurred when the patient’s marking runs parallel to the contour of the letter (see middle-right example). (**B**) Patients were asked to cross out all small stars on the page. A green dot indicates that a target star was detected as crossed out, while a red dot indicates that it was not detected as crossed out. The left column depicts examples in which target stars were falsely detected as crossed out. The right column depicts examples in which target stars were incorrectly detected as not crossed out, despite being correctly marked by patients. These errors can result from low-quality prints introducing noise (see bottom left) or from the marking of another star being nearby (see top left). Failures to detect crossed-out stars were often caused by markings running along the star’s outline (see top right).

Figure 10 illustrates the type of errors that occurred in the letter cancellation subtest, which fell into two categories: false positives (CANDO incorrectly identified an uncrossed target as crossed out) and false negatives (failing to detect a correctly crossed-out target). In most cases, only a single error occurred per scan. Seven scans contained false positives, mostly caused by low print quality or close proximity between letters. Another seven contained false negatives, caused by markings that followed the contours of the letters.

#### 3.2.3. Star Cancellation

After applying the scan inclusion criteria, scans from 78 patients were eligible for analysis. The average number of detected crossed-out targets (i.e., the small stars) was M = 43.79 (Mdn = 50; SD = 12.55) compared to the ground truth of M = 43.96 (Mdn = 50; SD = 12.69). A Kendall tau correlation between CANDO and ground-truth counts yielded a significant correlation of τ = 0.953, *p* < 0.001 (Figure 9C). Of the 78 scans, CANDO failed to detect existing markings or detected nonexistent markings in 14 scans. In most of these scans, CANDO produced only one error. Two, three, or four errors per scan were only found once each. Agreement between the manually established ground truth and CANDO was nevertheless extremely high (ICC(A,1) = 0.998, 95% CI [0.997, 0.999]). Exact agreement was observed in 82.05% of cases, and 94.87% of evaluations differed by no more than one target. The mean absolute difference was 0.27 targets.

Of the 14 scans with errors, ten contained false negatives and four contained false positives. False positives resulted from either another marking being nearby (e.g., top- and middle-left example in Figure 10B) or from printing/scanning noise on the scan (e.g., bottom-left example in Figure 10B). False negatives were, in most cases, markings that ran almost perfectly along the star outline such that they are barely detectable (e.g., top- and middle-right example in Figure 10B). In one case, a false negative resulted from a very faint pen trace (e.g., bottom-right example in Figure 10B).

#### 3.2.4. Line Bisection

After applying the scan inclusion criteria, 77 patient scans were included in the analysis. Unlike in the previous subtests, the values represent the sum of deviations from the center, calculated on a 0- to 3-point scale per line. The average number of points calculated by CANDO was M = 6.64 (Mdn = 8; SD = 2.83) compared to the ground truth of M = 6.83 (Mdn = 8; SD = 2.78). A Kendall tau correlation between the CANDO rating and the true values revealed a significant correlation of τ = 0.918 (*p* < 0.001) (Figure 9D). Agreement between the ground truth and CANDO was very high (ICC(A,1) = 0.946, 95% CI [0.913, 0.967]). Exact agreement was observed in 95.13% of cases, and all remaining evaluations differed by no more than one point. The mean absolute difference was 0.19 points.

Only three scans contained errors. These errors were caused by noise on the print/scan near the lines to be bisected, which was erroneously detected as bisection markers.

#### 3.2.5. Star Copying

**Figure 11:**
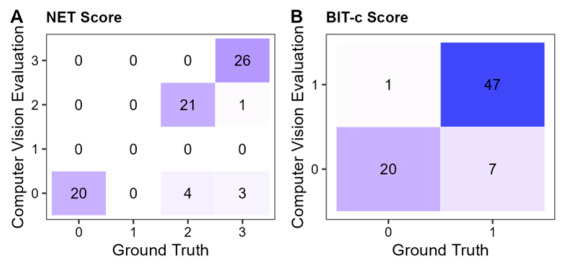
Agreement between CV detection and true value for (**A**) star-copying NET and (**B**) star-copying BIT-c. Note. Findings from the star-copying subtest. CANDO scores are plotted against ground truth using a heat matrix display. The darker the purple, the more cases in a given cell. This color gradient is fixed across the two plots. (**A**) presents the results for the NET, while (**B**) presents the results for the BIT-c.

In total, 75 patient files met the scan inclusion criteria and were included in the analysis. Within the NET, this subtest is evaluated on a scale from 0 to 3, which makes it difficult to evaluate the performance of CANDO on the basis of traditional descriptive statistics and correlations. We therefore calculated Cohen’s kappa to assess agreement between the CANDO rating and the manual rating: kappa = 0.840, z = 10.40, *p* < 0.001 with a total agreement of 89.33% (Figure 11A). According to Landis and Koch [45], kappa values above 0.80 suggest almost perfect agreement.

For the BIT-c version, a score of one or zero is given, depending on the completeness of the drawing. The same patient scans were used, and we calculated Cohen’s kappa for the BIT-c score to measure the agreement between CANDO and ground truth. Agreement was slightly lower than for the NET score: kappa = 0.757, z = 6.67, *p* < 0.001, but overall accuracy remained the same at 89.33% (Figure 11B). Kappas between 0.61 and 0.80 can be interpreted as in substantial agreement [45].

**Figure 12:**
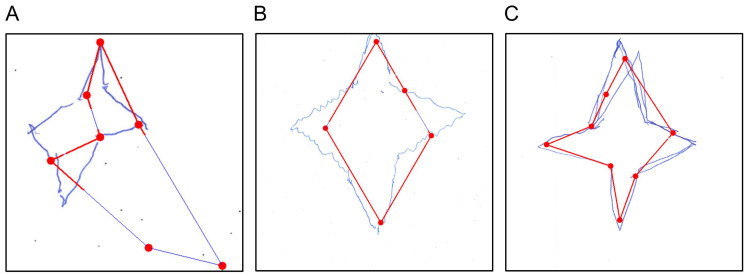
Example errors in the star-copying subtest. Note: Patients were asked to copy the drawing of a star. Red dots show the detected corners of the patient’s drawing, while the red lines reflect the idealized star derived from these corners. Errors occurred due to printing/scanning noise (**A**), tremor-induced waviness and gaps (**B**), or corrections added by the patient (**C**).

CANDO and ground truth disagreed in eight scans. Typically, CANDO assigned a score of zero where ground truth assigned a non-zero score. These discrepancies arose for several reasons: (1) substantial printing/scanning noise (Figure 12A; three cases); (2) tremor-induced waviness and gaps in the patient’s drawing (Figure 12B; one case); or (3) corrections added by the patient after the initial attempt (Figure 12; one case).

#### 3.2.6. Diamond Copying

**Figure 13:**
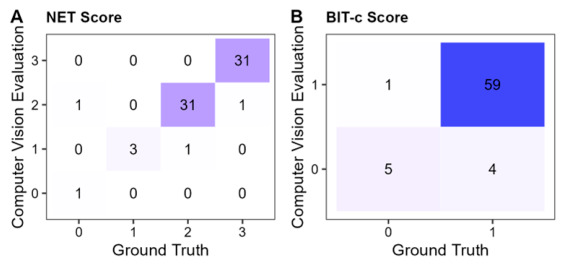
Agreement between CV detection and true value for (**A**) diamond-copying NET and (**B**) diamond-copying BIT-c. Note: findings from the diamond-copying subtest. CANDO scores are plotted against ground truth using a heat matrix display. The darker the purple, the more cases in a given cell. This color gradient is fixed across the two plots. (**A**) presents the results for the NET, while (**B**) presents the results for the BIT-c.

Based on the inclusion criteria, 69 patient files qualified for the analysis. In line with the analysis of the star-copying subtest, we calculated Cohen’s kappa to measure the agreement between CANDO and ground truth for the NET score and the BIT-c score. We found high agreement for the NET score with kappa = 0.923, z = 9.24, *p* < 0.001, and a total agreement of 95.65% (Figure 13A). For the BIT-c score, the analysis yielded kappa = 0.628, z = 5.35, *p* < 0.001, with a total agreement of 92.75% (Figure 13B).

**Figure 14:**
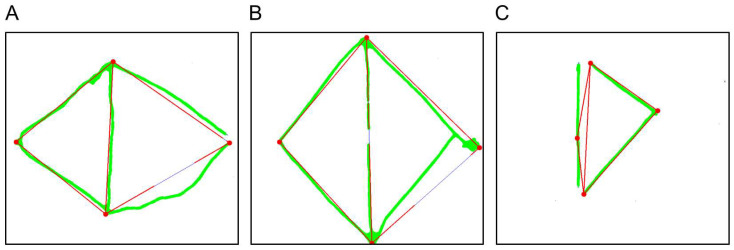
Example errors in the diamond-copying subtest. Note. Patients were asked to copy the drawing of a diamond. Red dots show the detected corners of the patient’s drawing, while the red lines reflect the idealized diamond derived from these corners. This is overlaid on top of the patient’s drawing, which is shown in green. Errors occurred due to very round contours deviating from the idealized template (**A**), patient corrections extending beyond the original drawing’s contours (**B**), or gaps and missing parts of the drawing (**C**).

As can be seen in Figure 13B, when scoring was performed in line with the instructions from the BIT-c manual, CANDO yielded five incorrect evaluations, three of which also produced errors under the NET scoring criteria. We examined these drawings to identify the sources of error. In one example, the bottom-right line was very curved, and since CANDO overlays an idealized diamond based on the detected corners, the curved line was not detected and the drawing was thus classified as incomplete (see left example in Figure 14A). In another example (Figure 14B), the patient initially drew the top-right line too long and corrected it when adding the bottom-right line. Because CANDO scales its idealized template to the outermost corners of the patient’s drawing, this correction shifted the reference points outward, producing a template larger than the intended drawing. The resulting mismatch between the template and patient contours caused CANDO to classify the drawing as incomplete. In another case (Figure 14C), the left half of the diamond was missing, which alone would not typically cause an error. However, a gap between the midline and the top and bottom corners caused CANDO to misidentify the midline as the left half of the diamond, leading it to conclude that no midline was present.

#### 3.2.7. Influence on Diagnosis

Finally, we consider the impact of CANDO’s errors on neglect diagnosis. The BIT-c provides diagnostic cut-off values for the subtest and the total score. Here, we focus on individual subtests and examine how the described errors would have influenced a diagnosis based solely on the one subtest in question. This analysis is limited to three subtests: line crossing, star cancellation, and line bisection. We did not examine the full set of stimuli for the figure-and-shape-copying subtest and therefore cannot apply the BIT-c cut-off for this subtest.

To characterize diagnostic agreement, we additionally examined confusion matrices comparing neglect classifications derived from the ground-truth scores and the corresponding CANDO-derived scores. For the line-crossing subtest, CANDO achieved perfect diagnostic agreement (accuracy = 100%, sensitivity = 100%, specificity = 100%). Similarly high classification performance was observed for the remaining subtests: letter cancellation (accuracy: 96.36%, sensitivity = 100%, specificity = 92.86%), star cancellation (accuracy = 96.36%, sensitivity = 100%, specificity = 90.32%), and line bisection (accuracy = 96.88%, sensitivity = 100%, specificity = 94.44%). Across all subtests, CANDO did not fail to identify any neglect cases defined by the ground-truth classification, with all diagnostic disagreements reflecting false-positive, rather than false-negative classifications.

To complement this analysis, we conducted exploratory receiver-operating characteristics (ROC) analyses for the four subtests with available diagnostic cut-offs. For each subtest, the diagnosis derived from the manually established ground-truth score served as the reference classification, while the corresponding CANDO-derived score served as the predictor. These analyses therefore assess whether the automated score preserves the diagnostic information contained in the manually evaluated subtest score. CANDO showed excellent discriminative performance across subtests: line crossing—AUC = 1.00, 95% CI [1.00, 1.00]; letter cancellation—AUC = 0.987, 95% CI [0.963, 1.000]; star cancellation—AUC = 0.990, 95% CI [0.976, 1.000]; and line bisection—AUC = 0.972, 95% CI [0.928, 1.000].

Overall, CANDO errors would have led to a false-positive misdiagnosis in seven patients (6.93%). However, it is important to note that this misdiagnosis rate only holds if two unlikely conditions are met: (1) the diagnosis is based on a single subtest, which is considered poor practice, and (2) users do not review the analyzed scans for obvious errors.

Regarding the first condition: among the seven patients whose diagnosis would have differed if based on a single subtest, all had at least one additional subtest that was evaluated correctly by CANDO. Thus, for most of these patients, the incorrect result from one subtest would have been compensated by an accurate result from another. Only two patients (1.98% of the full sample; N = 101) presented cases where the diagnostic error could have been consequential. In the first case, the patient had completed only one additional subtest. Although this second subtest was evaluated correctly, the diagnosis would still rest on one correct and one incorrect result, leaving greater uncertainty. In the second case, the patient had three subtests, two of which were evaluated incorrectly, though only one error affected the diagnosis. The third subtest was evaluated correctly. Thus, in both cases, the primary consequence was not an incorrect diagnosis but rather increased diagnostic uncertainty.

Regarding the second condition, we refer the reader to Figure 10, Figure 12 and Figure 14, which illustrate examples of CV errors. These errors are readily apparent to the human eye and can be easily identified and corrected. This raises a broader question that we address in greater detail below: how should CANDO be integrated into the assessment procedure? Raw values for each incorrectly analyzed scan are available in Table S5 in the Supplementary Materials.

#### 3.2.8. Evaluation on an Independent Holdout Cohort

As an additional analysis, CANDO was evaluated on an independent set of eleven retrospectively selected patients from our outpatient clinic. The cohort had a mean age of 55.18 years (SD = 16.93 years), included three females, eight males and two patients with neglect, and lesions were located in the left (two patients) and right (seven patients) hemispheres. An additional two patients suffered from bilateral lesions. Unfortunately, not every subtest was conducted and not every filled-out test sheet was usable. The performance of CANDO was identical to the ground truth, with 100% (11 out of 11 files) in the line-crossing subtest, 100% (10 out of 10) in the line-bisection subtest, 100% (5 out of 5) in the star-cancellation task, 90% (9 out 10) for the NET and BIT evaluation of star copying, and 100% (11 out of 11) for NET and BIT evaluation of diamond copying.

#### 3.2.9. Agreement Between Human Raters and CANDO

To further examine the influence of the manually established ground truth on the reported CANDO performance, two experts (LB and DS) independently evaluated a subset of 33 complete patient files. We then compared agreement between the two expert raters as well as agreement between each rater and CANDO across all currently automated subtests (Table 3 and Table 4).

Agreement between the two expert raters was extremely high for the cancellation and line-bisection subtests. Exact agreement ranged from 87.88% to 96.97% (see Table 3), with intra-class correlation coefficient (ICC) values between 0.988 and 1.000, indicating excellent agreement [46]. Although exact agreement between CANDO and the raters was somewhat lower for the letter- and star-cancellation tasks (66.67–81.82%), disagreements were generally very small in magnitude, with mean absolute differences below one target in all cases (see Table 3). More than 93% of all cancellation and line-bisection evaluations differed by no more than a single point or target.

A different pattern emerged for the figure-and-shape-copying tasks (see Table 4). For the binary BIT-c scoring criteria, agreement remained high both between the two expert raters (κ = 0.891−1.00) and between CANDO and the individual raters (κ = 0.615−0.933). In contrast, agreement for the multi-level NET copying criteria was substantially lower between the two expert raters (κ = 0.375−0.678), particularly for the diamond-copying task. According to Landis and Koch [45], kappas between 0.21 and 0.40 should be interpreted as in fair agreement and between 0.41 and 0.60 as in moderate agreement. Thus, compared to all other tasks, the NET copying evaluation was relatively weak in agreement.

The discrepancy in weighted kappa values between the BIT-c and NET copying evaluations was particularly pronounced. To further examine the source of this difference, we recalculated the NET copying scores using only the shape and detail components, thereby excluding the arrangement criterion and focusing primarily on completeness rather than geometric distortions. This substantially increased both exact agreement and weighted kappa values, see rows “star copying (NET), arrangement excluded” and “diamond copying (NET), arrangement excluded” in Table 4. Thus, for both copying tasks, removing the arrangement score led to what is considered substantial agreement [45].

These findings suggest that variability in the NET copying evaluations was driven primarily by the arrangement criterion rather than by disagreements regarding the completeness of the drawings.

### 3.3. Interim Discussion

We developed a CV-based tool, CANDO, to automate and standardize the evaluation of patient responses on the BIT-c and NET. We found high correlations between CANDO and ground truth for the cancellation subtests and line-bisection subtest, ranging from τ = 0.918 to τ = 0.998. For the figure-and-shape-copying subtests (star and diamond), we found high agreement between CANDO and manual ratings, ranging from 89.33% to 95.65%. By design, test–retest agreement is 100%, as CANDO always treats the same scan in the same manner, and, provided the best-practice recommendations outlined in the general discussion are followed, rescanning of test sheets should not affect the evaluation. Processing a single patient takes approximately 1.5 min on a standard consumer-grade laptop (Intel Core i5, 16 GB RAM, Windows 10), though processing time may vary depending on the hardware used. Our analysis suggests that CANDO errors are rare, easy to spot, and mostly have little or no impact on the final diagnostic decision. In our view, CANDO therefore represents an advance over current assessment practices, which, as demonstrated in Part 1, produce ratings that vary both across and within raters.

## 4. General Discussion

In this manuscript, we pursued two goals. First, we aimed to examine the consistency and efficiency of the manual scoring process for the most relevant test battery for unilateral neglect: the BIT-c [11] and its German adaptation, the NET [13]. Second, we developed and evaluated an automated scoring system for these two test batteries. In short, we found that manual scoring leads to some inconsistencies, particularly for the figure-and-shape-copying subtest, and takes considerable time when performed conscientiously. We found that a computer vision (CV)-based system (CANDO) can substantially reduce the time needed to score the BIT-c (or NET), produce highly accurate and reliable scores, and provide easy access to measures that can improve the sensitivity of the tests. Below, we first discuss the key findings from each part, describe the implications of our findings, and provide specific recommendations for using CANDO.

In Part 1, we evaluated agreement between raters and within-rater consistency across repeated ratings for a subset of subtests from the BIT-c [11] and the NET [13]. Overall, diagnostic agreement based on a weighted BIT-c sum score was high, with only rare discrepancies in assigning neglect (N− vs. N+). The NET, which includes a graded severity classification, showed more variability. However, most diagnostic differences between raters involved only a shift of one severity category. Notably, there was substantial disagreement between diagnoses derived from the BIT-c vs. the NET, which we attribute to differences in the incidental weighting of the different subtests, particularly the relative influence of the representational drawing- and figure-and-shape-copying tasks. These subtests showed the lowest agreement, both between and within raters. In contrast, the line-bisection and cancellation subtests exhibited stronger agreement. Interestingly, neither collapsing the NET’s “mild” category into a no-neglect category nor employing the NET’s more detailed scoring criteria substantially improved rater agreement. While experts tended to demonstrate slightly higher consistency than naïve raters with and without practice, group differences were generally negligible.

These findings highlight that some components of neglect diagnostics—especially drawing and copying subtests—are prone to subjective interpretation, even when detailed scoring criteria are specified. The variability in scoring these tasks, together with the considerable time required for careful manual scoring, underscores the need for more standardized and efficient assessment procedures.

In Part 2, we evaluated the performance of an automated CV-based analysis system across a larger clinical dataset (N = 101 patients with acquired brain damage). CANDO performed robustly, especially on structured subtests like line bisection and line crossing. Errors in the cancellation subtests were rare and small in magnitude. Importantly, the system also demonstrated good performance on more complex drawing subtests (e.g., star and diamond copying), offering a consistent and objective scoring approach across all patients. The diagnostic impact of CANDO’s scoring errors was low: only seven out of the 101 patients would have received a different diagnosis if each subtest were considered in isolation—a scenario that would be further mitigated when multiple subtests are used together. These results support CANDO’s use as a tool to assist—but not replace—clinical judgment.

### 4.1. Problems with Manual Evaluation—Lack of Specificity and Subjectivity in Evaluating the Copying and Drawing Subtests

Manual evaluation of the BIT-c/NET has some drawbacks. Particularly for the representational drawing and copying subtests, the BIT-c evaluation criteria are vague. The manual states that “The scoring of this subtest is based on the completeness of the respective drawing (1–4). Failure to complete is defined as the omission of any major component of the drawing (examples are provided in the Appendix).” ([11], p. 11). This allows for a very open interpretation of what constitutes a major component and what counts as missing. However, the examples in the appendix cover only clear-cut cases and do not address edge cases or the idiosyncratic responses frequently encountered in clinical practice. The NET manual [13] provides more detailed scoring instructions, with separate points awarded for shape/gestalt, details (e.g., the four rays in the star, or the clock face and hands), and arrangement.

Unfortunately, the more specific evaluation criteria in the NET did not substantially improve rating consistency. As with the BIT-c, raters were left to develop their own operationalizations for the gestalt and arrangement scores. We illustrate these problems using the star-copying task. The original star has four points. If one point is missing, a score of 0 must be awarded (BIT: 0 for the entire subtest; NET: 0 for the gestalt criterion). However, when do we consider that point to be missing? What if only the very tip of the point is absent? A similar ambiguity arises for the NET arrangement criterion: in the template, the lines leading to the four points in the star are consistently inclined at the same angle. How much deviation from the template justifies withholding the arrangement score? The comparison between two raters and CANDO actually highlighted this issue, as the relatively low consistency between the two raters and one rater and CANDO, could be well explained by a different criterion for the arrangement of the drawing features.

This highlights a further issue: performance in the drawing/copying subtests reflects not just perceptual skills (of primary concern when testing for neglect) but also motor skills. How should raters disentangle these two sources of error? More generally, applying the scoring instructions to specific cases requires subjective judgments on the part of raters. These judgments will be based on implicit criteria that vary between raters and over time. While it is impossible to specify which criteria are correct, we can aim for consistency. CANDO locks in the specific criteria for each of those judgments and thus achieves perfect consistency across patients and time.

### 4.2. Minimum Acceptable Accuracy

Having discussed the limitations of manual evaluation, we now consider the accuracy of CANDO. Under a strict criterion—where only fully correct judgments were counted as accurate—CANDO’s accuracy ranged from 74.55% to 96.97%. When a small margin of error was permitted (up to 2.5% deviation from the maximum possible score in each subtest), accuracy increased to between 94.55% and 96.97%. This tolerance reflects minor scoring differences that are negligible in high-scoring tests, such as the star-cancellation subtest. Importantly, when accuracy was calculated at the diagnostic level (i.e., whether a judgment based on performance in a single subtest led to correct diagnosis), CANDO errors affected diagnosis in only seven patients, yielding an overall diagnostic accuracy of 93.07%.

A key question that follows is whether this level of accuracy is sufficiently high. Cabitza and colleagues [47] have proposed a framework for determining the minimum acceptable accuracy (MAA) of binary diagnostic criteria in medical AI systems (e.g., distinguishing between normal vs. abnormal cases, or improvement vs. no improvement). They argue that the MAA should be defined according to several factors: (1) the criticality of the task (e.g., whether the system is used for diagnosing life-threatening conditions, which would demand higher accuracy); (2) comparison with human performance levels; and (3) the acceptable margin of error in clinical practice [47].

Neglect is not a life-threatening condition, so accuracy requirements can be somewhat relaxed. Neglect, like many other neuropsychological disorders, can be classified as a chronic condition. For chronic conditions, higher error rates can be tolerated, since few clinical decisions are irreversible and repeat encounters with patients mean that individual diagnostic errors are likely to be corrected at a later stage. Furthermore, as demonstrated in Part 1, the human baseline against which a CV-based system must be judged is itself imperfect—even under ideal conditions, human raters make occasional scoring errors. In comparison to typical manual assessments, CANDO already performs well. However, we strongly recommend making use of the option to visualize the processing steps, especially the final one showing the full evaluation (see the README.md file for instructions).

### 4.3. Best-Practice Suggestions

**Figure 15:**
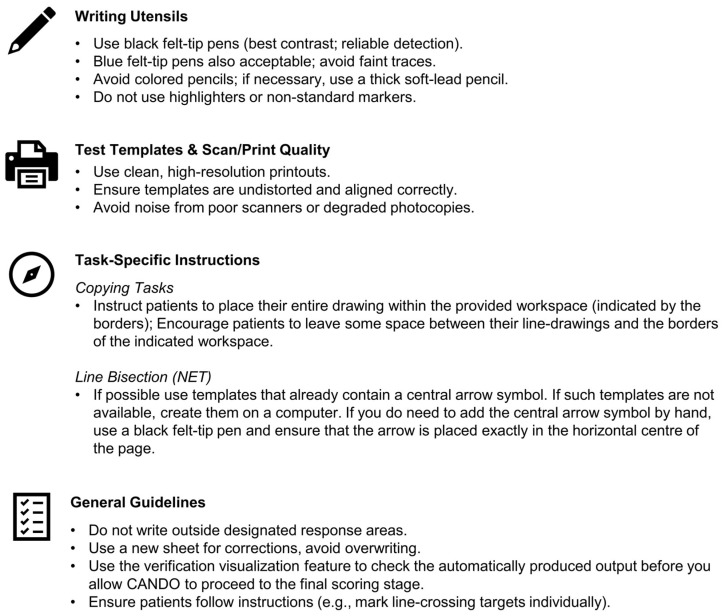
Best-practice suggestions.

To identify conditions that yield the best results with CANDO, we conducted additional controlled tests using different pen types, line widths, drawing styles, and print qualities. These tests informed a set of practical recommendations (summarized in Figure 15) that can help to minimize errors during test administration and document acquisition.

Our recommendations focus on four aspects of BIT-c/NET administration to ensure the most suitable input for CANDO analysis. First, we recommend using appropriate writing tools—ideally a black felt pen producing high contrast on paper. Pencils, thin pens, and some colored pens (especially red) are not recommended because they may reduce grayscale contrast and interfere with reliable contour detection. Second, we emphasize the use of high-quality, undistorted test sheets, free of substantial pixel noise. Third, we offer several subtest-specific recommendations, such as ensuring that drawings remain well within the frame in the figure-and-shape-copying subtest and that central, high-contrast arrows are added to the line-bisection subtest (for the NET). Finally, we recommend several general practices: corrections on the test sheet should be avoided in favor of using a new sheet, extraneous markings on the front page should be avoided, and users should take advantage of the verification feature to confirm the correctness of CANDO’s output. Together, these practices help minimize errors.

### 4.4. Limitations and Future Directions

Despite CANDO’s strong performance, several limitations arise from both the nature of the underlying clinical tasks and the constraints of classical CV methods. Below, we discuss these limitations alongside future opportunities for CANDO.

First, some BIT-c/NET subtests—most notably the representational drawing subtest (i.e., clock drawing), the Necker cube, and the flower drawing—are not yet automated. Our attempts to implement CV-based scoring for these subtests revealed that classical image-processing techniques struggle with the high variability and idiosyncratic response patterns frequently observed after stroke. As discussed earlier, these subtests are inherently difficult to interpret even for human raters, because errors may arise from a broad range of cognitive or motor impairments unrelated to neglect. This interpretive ambiguity limits the suitability of rule-based CV approaches and suggests that machine learning models, trained on sufficiently large annotated datasets, may be better positioned to capture the complex visuoconstructive features required for reliable scoring. At the same time, the diagnostic relevance of fully automating these subtests is doubtful for several reasons. First, the flower is similar to the star, and most patients with neglect who produce errors in the star can be expected to produce comparable errors in the flower. Second, performance in Necker cube copying and clock drawing is vulnerable not just to neglect-related problems but is also affected by difficulties in 3D perception and visuoconstruction, as well as other non-perceptual cognitive deficits [48] and level of verbal intelligence [30]. Indeed, the clock-drawing test is frequently used to screen for signs of dementia, even in patients where no neglect deficits are suspected [49,50,51]. In sum, the three copying/drawing subtests not included in CANDO may generate little additional information or may even reduce the specificity of the neglect tests. For this reason, the omission of these three tasks may not necessarily constitute a major shortcoming of the current system.

A second limitation is that CANDO is subject to technical constraints that do not affect manual scoring. CANDO translates all content on a scanned page into pixel intensities and cannot distinguish intended marks from unintended ones, nor erroneous marks from deliberate corrections. Furthermore, faint pencil traces, warped or distorted prints, misaligned scans, or markings outside template boundaries can all reduce detection accuracy or prevent the program from completing the analysis. Many such errors, however, result from using non-optimal conditions during test administration. The dataset used in the present study was collected prior to CANDO’s development and was therefore created under such conditions. If the best-practice suggestions (see Figure 15) are followed, most of the errors described above can be prevented.

More generally, these issues reflect a broader limitation in handcrafted rule-based feature extraction approaches. Unlike learning-based systems that may adapt flexible to variable input, deterministic pipelines remain dependent on predefined geometric assumptions and explicitly implemented decision rules. Consequently, unusual drawing strategies, substantial deviations from print geometry, or non-standard acquisition conditions may reduce robustness because the system cannot dynamically infer alternative feature representations beyond the specified rules. At the same time, the present exclusion criteria inevitably limit the reported performance estimates to relatively standardizable and scannable cases. However, many excluded scans reflected preventable acquisition problems or conditions that complicate both automated and manual evaluation, e.g., multiple marking in the line bisection task. Future work should therefore more systematically examine how technically problematic scans can be identified automatically and actively flagged for manual review.

It should also be noted that the ground truth in Part 2 was based on the evaluation of a single human rater. While the iterative correction procedure was designed to enhance consistency and ensure correctness, some degree of subjectivity cannot be entirely excluded, particularly in light of the inter-rater variability observed in Part 1. To further examine this issue, we conducted an additional follow-up analysis comparing two expert raters and CANDO on a subset of patient files. Agreement between the two human raters, as well as between raters and CANDO, was extremely high for quantitative cancellation and line-bisection tasks and remained high for the binary BIT-c copying criteria. In contrast, lower agreement emerged for the multi-level NET copying evaluations, particularly for the arrangement criterion, suggesting that this aspect of the scoring procedure remains comparatively underspecified. Importantly, agreement between CANDO and individual raters for these tasks was comparable to, and in some cases exceeded, agreement between the two human raters themselves, indicating that disagreement primarily reflected differences in the interpretation of qualitative geometric distortions rather than instability introduced by the automated approach.

A further limitation concerns the generalizability of the present findings. Because Parts 1 and 2 were designed to address different research questions, they relied on different datasets and therefore do not permit direct large-scale comparison between human raters and automated evaluation on the same patient sample. In Part 1, composite cases were deliberately constructed to include diagnostically challenging response patterns in order to examine potential sources of disagreement rather than estimate their real-world frequency. In Part 2, CANDO was evaluated on a retrospective convenience sample collected primarily in rehabilitation settings. Although the overall dataset included scans from multiple sources and rehabilitation stages, the additional evaluation on an independent holdout cohort was conducted on a smaller subset, acquired under relatively standardized conditions from a single clinical center. Consequently, the present findings may not fully generalize to broader clinical environments characterized by greater variability in scanner hardware, acquisition procedures, handwriting styles, acute bedside assessment, or institutional administration practices. Future work should therefore validate the present findings prospectively in larger and more heterogenous multi-center cohorts. Our best-practice suggestions, however, also have the potential to reduce the number of future exclusions if followed.

Many of the limitations described above reflect a simple truth: a CV-based system will sometimes struggle with decisions that are easy for the human observer. Rather than aiming for a system that replicates everything a human rater can do, a more productive approach may be to capitalize on the features that give CANDO an advantage over human raters. Three such features are worth highlighting. First, CANDO is perfectly reliable: for a given scan, it will always deliver the same result. This is particularly valuable when the BIT-c or NET are used in a scientific context, where consistency and objectivity are paramount. Importantly, we do not envision CANDO as completely replacing expert clinical judgment, but rather as a semi-automated decision tool that allows clinicians to efficiently verify and, if necessary, correct its outputs. Because many CANDO errors are visually obvious and easily identifiable by human observers, future work should examine how such workflows influence rating speed, diagnostic confidence, and inter-rater agreement under realistic clinical conditions.

An additional follow-up analysis comparing agreement between two expert raters and CANDO further highlighted the interpretive ambiguity of the qualitative NET copying criteria. Further follow-up analyses showed that removing the arrangement component markedly increased interrater agreement, suggesting that this criterion is a major source of variability. Importantly, agreement between CANDO and individual raters for these tasks was comparable to, and in some cases, exceeded, the agreement observed between the two human raters themselves. This pattern suggests that disagreements primarily reflect differences in how qualitative geometric distortions are interpreted rather than instability introduced by the automated approach itself.

This leads to the second feature of a deterministic pipeline we would like to highlight. Importantly, because CANDO explicitly operationalizes these criteria, the underlying geometric thresholds can be adjusted transparently by the user depending on the intended application. For example, research context aiming to exclude even subtle neglect symptoms may favor stricter criteria, whereas clinical screening context may prioritize greater sensitivity by tolerating minor geometric distortions. This flexibility represents one advantage of deterministic rule-based approaches: the applied decision criteria remain transparent, reproducible, and directly modifiable depending on the intended application.

Third, CANDO can easily generate additional test parameters not included in the standard BIT-c/NET evaluation. Why would such additional parameters be useful? Unilateral neglect is fundamentally a side-specific disorder, characterized by a strongly lateralized pattern of errors. Yet the standard BIT-c/NET scoring procedure gives limited weight to this lateralization component. Several well-established measures, such as the center of cancellation [15] or the endpoint weighting bias [52,53] for line bisection, account for the lateralized nature of test performance and thereby increase the diagnostic specificity of the test results.

A major added value of the automated approach is that it provides a straightforward and effortless method to derive such lateralized indices across cancellation, copying, and line-bisection subtests. This capability enhances the interpretability of ambiguous performance patterns and increases both sensitivity and specificity in detecting neglect. In future work, extending the system to compute more refined lateralization metrics—such as automated endpoint weighting bias or spatial weighting of copying errors—may prove more diagnostically valuable than attempting to automate all remaining BIT-c/NET subtests.

Beyond these considerations, the translational potential of CANDO may be particularly relevant in both routine clinical practice and research settings. In clinical deployment, CANDO is best conceptualized not as a replacement for expert evaluation but as a decision-support tool that pre-scores patient files and allows clinicians to rapidly verify and potentially correct automated outputs. Because the scoring process remains fully transparent and auditable, clinicians can easily inspect how a given score was derived and retrospectively review archived patient scans, together with the corresponding automated evaluations. This may substantially reduce the time burden associated with routine scoring while simultaneously improving standardization, documentation quality, and confidence in the diagnosis.

In research contexts, these advantages may be even more important. Group assignment in neuropsychological neglect research often depends critically on highly consistent application of diagnostic criteria, as even small differences in scoring thresholds can substantially influence which patients are included or excluded from a study. Because CANDO applies identical criteria across all evaluations, it may improve reproducibility and reduce variability introduced by subjective interpretation. At the same time, the deterministic framework allows thresholds to be adjusted transparently depending on the intended purpose. Importantly, these adjustments remain explicit, reproducible, and fully auditable.

## 5. Conclusions

Taken together, our findings highlight both the strengths and limitations of current clinical practices in neglect assessment. While human ratings generally demonstrated high consistency, certain subtests—particularly representational drawing and figure-and-shape copying—remained vulnerable to subjective interpretation. CANDO offers an improvement in this regard, representing a concrete step toward standardization, especially for subtests where human agreement was low. In addition to improving consistency, such standardization also reduces the substantial time clinicians and researchers spend manually scoring patient files, allowing their expertise to be directed toward higher-quality interpretation rather than repetitive assessment work. We emphasize that the program was developed not to replace human evaluation but rather to support and expedite it. We anticipate that the combination of CANDO and human evaluation can meaningfully improve and accelerate neglect diagnosis, ensuring greater consistency and objectivity when the BIT-c/NET is used in research—particularly for the copying subtests.

## Figures and Tables

**Table 1:** Evaluation and differences in evaluation of the BIT-c and NET.

BIT-c		NET	
*Subtest*	*Evaluation*	*Changes in Subtest*	*Changes in Evaluation*
Line crossing	No. of crossed-out targets	Identical	Identical *
**Letter cancellation**	No. of crossed-out targets	Identical	Identical *
Star cancellation	No. of crossed-out targets	German distractor words	Identical *
**Figure and shape copying (a): star, Necker cube, flower**	Scoring (0 or 1) based on completeness per figure	Necker Cube is replaced by a diamond	Scoring on three criteria per figure: (1) gestalt, (2) details, (3) arrangement
Figure and shape copying (b): geometric shapes	Scoring (0 or 1) based on completeness per figure	Not included	Not included
**Line bisection**	deviation from true center based on scoring templates	Identical	Identical *
**Representational drawing: (a) clockface with numbers**, (b) simple drawing of a man or woman, (c) simple drawing of a butterfly	Scoring (0 or 1) based on completeness per figure	Parts (b) and (c) are not included	Scoring on three criteria per figure: (1) gestalt, (2) details, (3) correctness

Note. * Initial evaluation is identical to the BIT-c; raw values are then converted to standardized scores between 0 and 10. Subtests used in Part 1 of the present study are highlighted in bold font.

**Table 2:** Demographic data.

	All	N+	N−
*Total*	101	54	47
*Gender (% male)*	54.46	59.26	48.94
*Age (years)*	63.25	67.42	58.45
*Lesion site (% right)*	78.22	88.88	65.96
*Visual field defects (%)*	28.72	11.11	48.94

**Table 3:** ICC.

Subtest	Comparison	Exact Agreement (%)	Agreement Within ± 1 (%)	Mean Absolute Difference	ICC [95% CI]
Line crossing	LB vs. DS	96.97	96.97	0.06	0.998 [0.997, 0.999]
	LB vs. CANDO	93.94	96.97	0.09	0.998 [0.997, 0.999]
	DS vs. CANDO	90.91	93.94	0.15	0.997 [0.994, 0.999]
Letter cancellation	LB vs. DS	93.94	100.00	0.06	1.000 [0.999, 1.000]
	LB vs. CANDO	72.73	93.94	0.64	0.969 [0.938, 0.984]
	DS vs. CANDO	66.67	93.94	0.70	0.968 [0.938, 0.984]
Star cancellation	LB vs. DS	87.88	100.00	0.12	1.000 [0.999, 1.000]
	LB vs. CANDO	81.82	93.94	0.24	1.000 [1.000, 1.000]
	DS vs. CANDO	78.79	93.94	0.30	0.998 [0.996, 0.999]
Line bisection *	LB vs. DS	96.97	100.00	0.03	0.998 [0.997, 0.999]
	LB vs. CANDO	90.91	96.97	0.15	0.982 [0.964, 0.991]
	DS vs. CANDO	87.88	96.97	0.18	0.980 [0.961, 0.990]

Note. Agreement statistics for cancellation and line-bisection subtests based on a subset of 33 complete patient files independently evaluated by two expert raters (LB and DS) and CANDO. “Agreement within ± 1” indicates the percentage of evaluations differing by no more than one target (cancellation tasks) or one point (line bisection). ICC—intra-class correlation coefficient. * Lower agreement in the line-bisection task may partly reflect minor mismatches between the manual scoring template and the original test sheet, which particularly affect borderline cases defined by percentage cut-offs.

**Table 4:** Weighted Cohen’s Kappa.

Subtest	Comparison	Exact Agreement (%)	Mean Absolute Difference	Weighted Kappa
Star copying (NET)	LB vs. DS	45.45	0.67	0.678
	LB vs. CANDO	81.82	0.21	0.898
	DS vs. CANDO	42.42	0.82	0.547
Star copying (NET), arrangement excluded	LB vs. DS	81.82	0.24	0.747
	LB vs. CANDO	90.91	0.12	0.895
	DS vs. CANDO	75.76	0.36	0.633
Star copying (BIT)	LB vs. DS	100.00	0.00	1.000
	LB vs. CANDO	96.97	0.03	0.933
	DS vs. CANDO	96.97	0.03	0.933
Diamond copying (NET)	LB vs. DS	39.39	0.64	0.375
	LB vs. CANDO	84.85	0.15	0.813
	DS vs. CANDO	48.48	0.55	0.402
Diamond copying (NET), arrangement excluded	LB vs. DS	93.94	0.06	0.764
	LB vs. CANDO	90.91	0.09	0.615
	DS vs. CANDO	90.91	0.09	0.615
Diamond copying (BIT)	LB vs. DS	94.97	0.03	0.891
	LB vs. CANDO	93.94	0.06	0.766
	DS vs. CANDO	90.91	0.09	0.615

Note. Agreement statistics for figure-and-shape-copying subtests based on a subset of 33 complete patient files independently evaluated by two expert raters (LB and DS) and CANDO. Weighted Cohen’s kappa values were used because the NET copying criteria involve ordinal multi-level scoring categories.

## Data Availability

The code for CANDO itself is freely available for use under https://osf.io/qdfk9. Exemplary results can be found in the Supplementary Materials. The data that we used to evaluate conventional rating procedures and the performance of CANDO are not readily available because they consist of clinical diagnostic data subject to ethical and privacy restrictions to protect patient anonymity. In addition, the informed consent obtained from participants does not explicitly permit the public sharing of diagnostic data. Requests to access the datasets should be directed to the corresponding author.

## Supplementary Materials

The following supporting information can be downloaded at https://www.mdpi.com/article/10.3390/brainsci16050541/s1, Table S1: Repeated-Measures Correlation between First and Second Rating for the BIT-c; Table S2: Repeated-Measures Correlation between First and Second Rating for the NET.; Table S3: Sample description per subtest; Table S4: Function specifications; Text S1: Detailed technical implementation of automation tool; Table S5: Influence of CV-based mistakes on the diagnosis.

## Abbreviations

The following abbreviations are used in this manuscript:

AI	Artificial intelligence
AUC	Area under the curve
BIT	Behavioural Inattention Test
BIT-c	Conventional subtests of the BIT
BIT-b	Behavioral subtests of the BIT
CANDO	Computer-based Analysis of Neglect Deficits On paper
CI	Confidence interval
CoC	Center-of-cancellation
CV	Computer vision
ICC	Intraclass correlation coefficient
M	Mean
Mdn	Median
ML	Machine learning
NET	Neglect Test
N+	Neglect patients
N−	Non-neglect patients
ROC	Receiver operating characteristics
VFD	Visual field defects
