# Supplementary material for: Paper to Pixels: Enhancing Unilateral Neglect Assessment Using the New Computer Vision-Based Tool CANDO
Source: Brain Sci. 2026 May 21;16(5):541. doi: 10.3390/brainsci16050541 (PMC13204769; doi:10.3390/brainsci16050541)
Supplement: Supplementary file 1 [file brainsci-16-00541-s001.zip › brainsci-4295252-supplementary.pdf]

Supplemental Material

S.1 Repeated Measures Correlations: First and Second Rating

Table S1. Repeated Measures Correlation between First and Second Rating for the BIT-c.

Subtest	r	CI	adjusted p-value
Letter Cancellation	0.99	[0.99, 0.99]	< .001
Copying: Star	0.85	[0.81, 0.89]	< .001
Copying: Diamond	0.92	[0.90, 0.94]	< .001
Copying: Cube	0.90	[0.87, 0.93]	< .001
Copying: Flower	0.94	[0.92, 0.96]	< .001
Line Bisection	0.98	[0.98, 0.99]	< .001
Representational Drawing	0.98	[0.98, 0.99]	< .001

Note. All p-values are Bonferroni corrected.

Table S2. Repeated Measures Correlation between First and Second Rating for the NET.

Subtest	r	CI	adjusted p-value
Letter Cancellation	0.98	[0.98, 0.99]	< .001
Copying: Star	0.85	[0.81, 0.89]	< .001
Copying: Diamond	0.87	[0.85, 0.91]	< .001
Copying: Cube	0.91	[0.88, 0.93]	< .001
Copying: Flower	0.93	[0.91, 0.95]	< .001
Line Bisection	0.98	[0.97, 0.99]	< .001
Representational Drawing	0.98	[0.97, 0.99]	< .001

Note. All p-values are Bonferroni corrected.

S.2 Additional information regarding the sample

Table S3. Sample description per subtest.

	**Line Crossing**			**Letter Cancellation**			**Star Cancellation**			**Line Bisection**			**Star Copying**			**Diamond Copying**		
	All	N+	N-	All	N+	N-	All	N+	N-	All	N+	N-	All	N+	N-	All	N+	N-
total	66	38	28	55	39	16	78	47	31	64	37	27	75	43	32	69	37	32
gender (% male)	60.61	65.79	53.57	63.64	66.67	56.25	61.54	63.83	58.06	56.25	59.46	51.85	57.33	65.12	46.88	60.87	67.57	53.13
age	64.03	67.63	59.14	66.27	66.15	66.56	64.13	66.87	59.97	64.13	68.46	58.19	65.40	68.00	61.91	61.88	65.73	57.44
lesion side (% right)	72.73	84.21	57.14	83.64	87.18	81.25	82.05	91.49	67.74	76.56	86.49	62.96	77.33	86.05	65.63	75.37	86.49	62.50
VFD (%)	25.76	7.89	50.00	5.45	5.13	6.25	21.79	10.64	41.94	23.44	10.82	40.74	22.67	9.30	40.63	30.43	5.41	59.38

Note. Overview of patient samples for each subtest. VFD = presence of visual field defects.

S.3 Function Specifications

Table S4. Function specifications.

Subtest	Files	Function	Output	Output Details
line crossing	LineC.png, LineC.jpg, LineC.jpeg	diag_LineC.process_image(file_path, LineC_T_C1)	LineC_LS	targets crossed on the left
			LineC_RS	targets crossed on the right
			LineC	crossed-out targets
			LineC_SV	standard value
letter cancellation	LetC.png, LetC.jpg LetC.jpeg	diag_LetC.process_image(file_path, folder_name)	LetC_LS	targets crossed on the left
			LetC_RS	targets crossed on the right
			LetC	crossed-out targets
			LetC_SV	standard value
star cancellation	StarC_de.png, StarC_de.jpg, StarC_de.jpeg, StarC_en.png, StarC_en.jpg, StarC_en.jpeg	diag_StarC.process_image(file_path, folder_name)	StarC_LS	targets crossed on the left
			StarC_RS	targets crossed on the right
			StarC	crossed-out targets
			StarC_SV	standard value
copying: star	Copy_de.png, Copy_de.jpg, Copy_de.jpeg,	diag_CopyStar.process_image(file_path)	CopyStar_S	NET shape score
			CopyStar_D	NET detail score
			CopyStar_A	NET arrangement score
			CopyStar_NET	overall NET score
			CopyStar	BIT-c score
			CopyStar_SV	standard value (NET)

	Copy_en.png, Copy_en.jpg, Copy_en.jpeg			
copying: diamond	Copy_de.png, Copy_de.jpg, Copy_de.jpeg	diag_CopyDiamond.process_image(file_path)	CopyDiamond_S	NET shape score
			CopyDiamond_D	NET detail score
			CopyDiamond_A	NET arrangement score
			CopyDiamond_NET	overall NET score
			CopyDiamond	BIT-c score
			CopyDiamond_SV	standard value (NET)
			CopyDiamond_hierarchical	NET hierarchical score
line bisection	LineB.png, LineB.jpg, LineB.jpeg	diag_LineB.process_image(file_path)	LineB_T	points top line
			LineB_M	points middle line
			LineB_B	points bottom line
			LineB	total score
			LineB_SV	standard value

Text S1. Detailed technical implementation of the automation tool

Technical implementation: Line crossing. In the line crossing task, patients must cross out all lines on the test sheet. For the evaluation, the number of lines the patient crossed out is counted. Our analysis pipeline produces a report that includes the conventional total score (*LineC*), left/right counts (*LineC_LS*, *LineC_RS*), and the NET standard value (*LineC_SV*).

Each patient scan is converted to grayscale and binarized to isolate pen marks from the background. Edge detection with hysteresis is then applied to emphasize true line edges while suppressing background texture, followed by denoising and morphological closing (dilation then erosion) to bridge small gaps without thickening the marks. A final binarization step completes preprocessing.

After preprocessing, contours are extracted and reduced to centroids. Only plausible marks are retained, and border artifacts are filtered out to avoid scan-induced detections. To

prevent double-counting when a single crossed line fragments into multiple blobs, nearby centroids are merged via density-based clustering. The small printed arrow on the sheet is then isolated and used to rotate the page to a canonical orientation, and all merged centroids are rotated accordingly to yield the final processed scan.

Crossed lines are then detected by comparing these centroids with the precomputed template targets: a template target is considered "hit" if a patient centroid falls within a small neighborhood of it. The four central demo targets (used by the examiner to demonstrate the task) are excluded by design.

Scoring proceeds by partitioning detections into left and right halves, yielding separate left and right counts whose sum gives the total count (maximum 36). The NET standard value is then assigned via the manual's lookup table (e.g., 35–36 maps to 10.0). All numeric thresholds (e.g., area ranges, merge radius, and midline margins) were empirically tuned to be robust to scan noise, pen pressure, and incomplete crossings.

Technical implementation: Letter cancellation. In the letter cancellation subtest, the number of crossed-out Es and Rs within a structured array is counted. In addition to the conventional total score (*LetC*), our pipeline reports separate left/right counts (*LetC_LS, LetC_RS*) and the NET standard value (*LetC_SV*).

Each patient scan is first normalized (resize, grayscale) and denoised before binarization to isolate high-contrast markings. As in the line crossing task, contours are used to localize the small printed arrow, which provides an orientation cue for aligning the page. The oriented image is then cropped to remove noisy margins and reprocessed (denoise, threshold) to produce a clean mask of the text block.

In order to detect the Es and Rs, we first identify the four corners of the text block by locating the black pixel nearest to each image corner and then shifting these points slightly outward to ensure full coverage. A perspective transform maps the block to a normalized canvas, after which the text is split into five equal horizontal lines. The positions of all Es and Rs are predefined by column indices (out of 34 per line). Using these indices, we extract a small image region centered on each target letter and record its metadata (ID, letter type, grid coordinates, etc.).

Each target letter image is then standardized and processed prior to detecting whether or not it is crossed out. We first grayscale and binarize each region, then detect contours and keep only the ones whose centroids lie near the region center to suppress interference from neighboring letters. After a second grayscaling and binarization, we select the largest remaining contour as the target letter, compute its centroid, and recenter it on a fixed frame size. Finally, we invert the image back to a dark-on-light representation, yielding a standardized letter image for classification.

Using this standardized target letter image, we then determine whether or not the letter is crossed out based on three different checks. First, a border-proximity check flags a crossing if any contour reaches near the top or bottom of the standardized frame, consistent with a slash traversing the letter. The second check searches for child contours which can be indicative of additional holes in the letter introduced by slashes. The final check specifically looks for the presence of a diagonal line within letter-specific regions of interest. It applies a probabilistic Hough line transform and accepts a candidate if its slope falls within empirically chosen ranges. A letter is marked as crossed if any of these checks succeed. The scoring proceeds almost identically to what is done for the line crossing task above.

Technical implementation: Star cancellation. In the star cancellation subtest, the number of crossed-out small stars within an array of small and large stars, as well as distractor letters and words, is counted (*StarC*). Additionally, the pipeline provides the number of targets crossed out on the left and right (*StarC_LS, StarC_RS*), along with the standard value (*StarC_SV*) in accordance with the NET manual.

Each patient scan is first converted to grayscale, denoised, and binarized, then lightly eroded to suppress speckle and scanning artifacts while preserving the underlying pen strokes. Contours are then extracted to locate the small printed arrow which again allows for orienting the page. The scan is then cropped to remove the unnecessary outer frame and reprocessed (denoise, threshold) to produce a clean mask of the array. To normalize geometry across scans, four reference points on the page are identified and used for a perspective transform onto a fixed template canvas. Small target stars are then isolated using predefined template coordinates (slightly varied depending on BIT vs. NET layout), omitting the two central small stars that are not clinically scored. Using these coordinates, we extract a small image region centered on each target star and record its metadata (ID, grid coordinates, etc.).

Similar to the letter cancellation task, each target star image is first standardized before classifying it as crossed out or not. Within a small window centered on the template coordinate, we binarize and detect contours, retaining only those whose centroids lie near the window center to suppress interference from neighboring symbols. We then re-binarize, select the largest remaining contour as the star, and compute its centroid. The star's topmost pixel provides an orientation cue to rotate the window such that the star is upright. Finally, we translate the star to the window center, yielding a normalized star image suitable for classification.

We classify a star as crossed if it triggers any of the following seven checks, each targeting a different way a pen stroke can alter the star's geometry. (1) We first check whether edge contours approach the window boundary, which is consistent with a stroke extending beyond the star. (2) We then skeletonize the star and count merged endpoints, flagging the star as crossed if there are more than five. (3) Next, we apply Harris corner detection on a lightly eroded image and merge nearby detections. If the number of merged corner points differs from five, we flag the star as crossed. (4) As an additional geometric cue, we test for slant by comparing the vertical alignment of the leftmost and rightmost pixels in the upper and lower portions of the window. A large offset is consistent with a diagonal crossing stroke. (5) We then count the number of black pixels in the center of the window, since a sufficiently high count confirms a crossing through the star's interior. (6) Next, we check the symmetry of the star's upper triangle by comparing the distances from the topmost point to the leftmost and rightmost extremes; if these distances differ beyond a small tolerance, the shape is likely distorted by an overlaid stroke and the star is flagged as crossed. (7) Finally, we verify the location of the bottom middle vertex of the star as a final sanity check on the star's geometry. Again, the scoring proceeds almost identically to what is done for the line crossing and letter cancellation tasks above.

Technical implementation: Copying star. In the figure and shape copying subtest, patients have to copy simple templates from the left side of a sheet of paper to the right. Specifically, in this part, this is a simple four-pointed star. The drawing is then evaluated based on its completeness (BIT, *CopyStar*) or on the closedness of the shape, the presence and arrangement of details (NET, *CopyStar_S, CopyStar_D, CopyStar_A, CopyStar_NET*). Again, a standard value is provided (*CopyStar_SV*)

In the star copying task, a patient reproduces a four-pointed star from a printed template and is scored on three criteria: shape, detail, and arrangement. Our pipeline reports these three component scores, a total score and its corresponding NET standard value, and geometric measures including internal angles, areas, and edge lengths.

The first step is to isolate the star drawing from the patient scan, which occupies the top-right corner of the page. After resizing to a standard resolution and converting to grayscale, Harris corner detection is applied and nearby detections are merged to recover stable grid intersections. Two consistent intersections from the printed guide layout serve as landmarks, and their coordinates define a bounding rectangle around the star region. That rectangle is slightly tightened and warped via a perspective transform to yield a standardized crop of the star drawing.

Next, the cropped region is denoised and binarized and Harris corner detection is run again to recover the star's vertices. We adapt the Harris block-size parameter to the star's span to keep corner detection stable across varying drawing sizes. Nearby detections are merged into stable corner points and outliers are filtered out. If too many corners remain, the closest pair is iteratively merged until the expected count of eight is reached. The final set of corners is sorted in a consistent clockwise order starting from the topmost one.

To evaluate whether the drawing actually traces a star, we connect these ordered corners to form an ideal outline and use it as a template for the expected geometry. This works because the corners capture the intended structure even when the drawn strokes are uneven or broken. For each edge of the ideal outline, we sample points along the edge and scan a perpendicular segment within a fixed tolerance band to look for ink. If any ink is found along that segment, the sample point counts as a hit. The fraction of hits along each edge is then computed, and an edge is marked as present when that fraction exceeds a minimum threshold. The result is a summary of which star edges are actually drawn.

From this edge summary, scoring proceeds in three parts. (1) The shape score checks whether all edges are present and whether the radial geometry is consistent: the tip corners must sit at roughly equal distances from the center, the inner corners must do the same at a shorter radius, and the gap between those two radii must fall within a meaningful range relative to the figure's span. (2) The detail score requires the shape score to pass and confirms that

exactly eight corners were detected, indicating a complete four-pointed star was drawn. (3) The arrangement score, which in turn requires the detail score, evaluates the drawing's symmetry and orientation: the vertical and horizontal spans must be roughly equal, the tips must be vertically and horizontally aligned, and the quadrilateral formed by the inner corners must have near-equal sides and near-right angles. Each criterion contributes one point, giving a total score from 0 to 3 that is mapped to a NET standard value via the manual's lookup table. Beyond scoring, the pipeline also extracts and reports geometric measures of the drawing, including all internal angles, the absolute vertical and horizontal axis angles, the areas of the left, right, top, and bottom halves, and the lengths of all edges.

Technical implementation: Copying diamond. Similar to the previous subtest described above, patients are asked to copy a figure, this time a diamond with a midline. For the BIT, the drawing is evaluated for completeness (*CopyDiamond*). For the NET, the closedness of the shape, the presence of details (i.e., midline), and the arrangement of angles are evaluated (*CopyDiamond_S, CopyDiamond_D, CopyDiamond_A, CopyDiamond_NET, CopyDiamond_SV*).

As in the star copying task, the first step is to isolate the diamond drawing from the patient scan, which occupies the middle-right area of the page. The same landmark-based cropping procedure is applied to yield a standardized crop of the diamond drawing. The cropped region is then converted to grayscale and passed through Canny edge detection, followed by denoising and morphological closing (dilation then erosion) to consolidate fragmented strokes into continuous contours. A Hough line transform then extracts straight line segments from the result. These segments are filtered by starting from the line closest to the image center and iteratively absorbing any nearby segments, discarding distant fragments unlikely to belong to the diamond.

From the surviving line segments, the program constructs an ideal diamond by first identifying the drawing's four extreme points (topmost, bottommost, leftmost, and rightmost) to determine the diamond's vertices. While the top and bottom vertices can be taken directly, the left and right corners are sometimes left open, particularly in drawings by patients with unilateral neglect. To account for this, the program checks whether each lateral extreme point has ink both above and below it. If it does, the corner is considered closed and that point is taken as the vertex. If not, the program extends the upper and lower edges until they intersect, using that point as the vertex. These four vertices define the ideal diamond outline, and a fifth

edge connecting the top and bottom vertices represents the vertical bisector. Each of these five edges is then validated against the patient's ink using the same perpendicular-scan approach as in the star copying task, producing a summary of which diamond edges are actually drawn.

From this edge summary, scoring proceeds in three parts. (1) The shape score checks whether all four outer edges of the diamond are present. (2) The detail score checks whether the vertical bisector is present. (3) The arrangement score checks whether the corner angles are consistent with the expected diamond geometry, flagging deviations beyond an acceptable tolerance. Each criterion contributes one point, giving a total score from 0 to 3 that is mapped to a NET standard value via the manual's lookup table. Beyond scoring, the pipeline also extracts geometric measures comparing the patient's drawing to the ideal diamond, including the four corner angles, the absolute vertical and horizontal axis angles, area ratios comparing the patient's left and right halves to the ideal halves, edge length ratios comparing each of the patient's four edges to the corresponding ideal edge, and the ratio of the vertical to horizontal span.

Technical implementation: Line bisection. In this subtest, patients are asked to mark the midpoint of three horizontal lines. The deviation from the midpoint is then evaluated for each line (*LineB_T, LineB_M, LineB_B*), attributing 0, 1, 2, or 3 points per line, with higher scores implying better performance. The final score is the sum of the points across the three lines (*LineB, LineB_SV*).

Each scan is first converted to grayscale and binarized, then passed through a multi-stage preprocessing pipeline using edge detection, denoising, and morphological expansion to emphasize thin pen marks while suppressing background texture. This process yields a high-contrast mask from which contours are extracted. We compute a centroid for each contour and merge nearby contours by centroid proximity to collapse fragmented strokes into single structures. The merged contours are then filtered to isolate only task-relevant markings: scan-border artifacts are discarded, the small printed arrow is isolated, and the remaining large contours corresponding to the target bisection lines are retained using size and border-distance criteria. To facilitate subsequent analysis, these retained line contours are simplified into lower-complexity polygonal approximations, and the page is finally rotated into a canonical orientation using the arrow contour as a fiducial.

For each of the three bisections, we estimate the left endpoint, right endpoint, and the patient's bisection mark directly from contour geometry. Specifically, we collect the most extreme contour points on all four sides and cluster these points into three groups; the leftmost and rightmost clusters define the line endpoints, while the central cluster corresponds to the bisection mark. The bisection mark's vertical coordinate is then set to the midpoint of the recovered endpoint heights for better alignment with the inferred line. The three bisections are then ordered by vertical position to correspond to the top, middle, and bottom lines.

Scoring is performed line-by-line by first computing each line's estimated midpoint from its recovered endpoints, then evaluating the bisection's horizontal offset within three nested tolerance bands centered at that midpoint (with band widths set as fixed proportions of the line length). The patient's bisection receives 1, 2, or 3 points based on the narrowest band it falls within (or 0 if it falls outside all bands) and is labeled as left- or right-biased depending on which side of the midpoint it lies. Scores are summed across the three lines (maximum 9) and mapped to the NET standard value via the manual's lookup table.

S.5 Influence on the diagnosis

Table S5. Influence of CV-based mistakes on the diagnosis.

Subtest	Patient	CV Score	CV Diagnosis	Ground Truth	Diagnosis	Agreement
line crossing	NHL	24	N+	26	N+	yes
	OBL	33	N+	34	N+	yes
letter cancellation	AVL	30	N+	29	N+	yes
	BJL	11	N+	10	N+	yes
	FQL	35	N-	36	N-	yes
	GBL	24	N+	27	N+	yes
	GZL	20	N+	19	N+	yes
	**HUL**	**32**	**N+**	**36**	**N-**	**no**
	KNL	39	N-	35	N-	yes
	KXL	22	N+	23	N+	yes
	MEL	36	N-	37	N-	yes
	OBL	25	N+	22	N+	yes
	QLL	40	N-	39	N-	yes
	QYL	39	N-	40	N-	yes
	**VHL**	**25**	**N+**	**36**	**N-**	**no**
	YIL	9	N+	8	N+	yes
star cancellation	BWD	53	N-	54	N-	yes
	GEL	25	N+	24	N+	yes
	IJL	53	N-	52	N-	yes
	LGD	53	N-	54	N-	yes
	MCL	35	N+	34	N+	yes
	OKL	22	N+	21	N+	yes
	QLK	44	N+	45	N+	yes
	**QLL**	**51**	**N+**	**52**	**N-**	**no**
	QPL	53	N-	54	N-	yes
	**SRL**	**50**	**N+**	**54**	**N-**	**no**
	UBK	44	N+	45	N+	yes

	VHL	41	N+	43	N+	yes
	**XFD**	**51**	**N+**	**54**	**N-**	**no**
	ZAL	44	N+	45	N+	yes
line bisection	**GAL**	**6**	**N+**	**9**	**N-**	**no**
	**LAL**	**3**	**N+**	**9**	**N-**	**no**
	OKL	3	N+	6	N+	yes

Note. Changes in scoring due to CV-based errors for each of the subtests. Rows highlighted in bold represent patients for which the diagnosis would change when applying the BIT cut-off score, also specified by the agreement column. There were no changes in diagnosis if based on the line crossing subtest, just two errors which did not affect the diagnosis ($N_{\text{line crossing}} = 66$). 14 patients had at least one error in the letter cancellation subtest, the diagnosis changed for two patients only (3.64%, $N_{\text{letter cancellation}} = 55$). 15 patients had at least one error in the star cancellation subtest, the diagnosis changed for three patients (3.85%, $N_{\text{star cancellation}} = 78$). Three patients had a falsely evaluated line bisection subtest, which affected the diagnosis in two cases (3.13%, $N_{\text{line bisection}} = 64$).
